# Deconvolution of clinical variance in CAR-T cell pharmacology and response

**DOI:** 10.1038/s41587-023-01687-x

**Published:** 2023-02-27

**Authors:** Daniel C. Kirouac, Cole Zmurchok, Avisek Deyati, Jordan Sicherman, Chris Bond, Peter W. Zandstra

**Affiliations:** 1 Notch Therapeutics, Vancouver, BC Canada; 2 School of Biomedical Engineering and Michael Smith Laboratories, University of British Columbia, Vancouver, BC Canada

**Keywords:** Computational models, Data integration, Predictive markers, Transcriptomics, Predictive medicine

## Abstract

Chimeric antigen receptor T cell (CAR-T) expansion and persistence vary widely among patients and predict both efficacy and toxicity. However, the mechanisms underlying clinical outcomes and patient variability are poorly defined. In this study, we developed a mathematical description of T cell responses wherein transitions among memory, effector and exhausted T cell states are coordinately regulated by tumor antigen engagement. The model is trained using clinical data from CAR-T products in different hematological malignancies and identifies cell-intrinsic differences in the turnover rate of memory cells and cytotoxic potency of effectors as the primary determinants of clinical response. Using a machine learning workflow, we demonstrate that product-intrinsic differences can accurately predict patient outcomes based on pre-infusion transcriptomes, and additional pharmacological variance arises from cellular interactions with patient tumors. We found that transcriptional signatures outperform T cell immunophenotyping as predictive of clinical response for two CD19-targeted CAR-T products in three indications, enabling a new phase of predictive CAR-T product development.

## Abstract (web summary)

A model of T cell differentiation control deconvolutes biological mechanisms underlying clinical variance.

## Main

Chimeric antigen receptor T cells (CAR-Ts) have shown remarkable activity in the treatment of B cell malignancies^1^. With six approved therapies and hundreds in clinical development for other hematological and solid tumors, genetically engineered T cells represent a therapeutic modality changing the drug development landscape^2^. However, T cells bring unique challenges to therapeutic development. These so-called ‘living drugs’ proliferate, differentiate, actively traffic between tissues and engage in two-way communication with the patient immune system. The resultant pharmacology is different from that of small molecules or biologics, as there is little relationship between administered dose and exposure^2^.

The cellular kinetics (pharmacokinetics) of circulating CAR-Ts are characterized by three distinct phases: initial expansion, followed by a rapid contraction and then slow, long-term decay^3^. The degree of cell expansion (Cmax) and long-term exposure (area under the curve (AUC)) vary widely among patients (approximtely three orders of magnitude) and are predictive of both efficacy (tumor size reduction) and toxicity^4^. However, the product-intrinsic and host-intrinsic factors mediating this pharmacology remain poorly defined. An empirical, non-linear mixed-effects model was developed to quantify the pharmacokinetics of Kymriah (tisagenlecleul, CTL019)^5^ and provided as part of the biologics license application (BLA)^4^. This formulation has proven applicable to multiple other CAR-T therapies in a variety of indications^6^ and has been adopted by the FDA for benchmarking^7,8^. Although a useful tool for quantifying clinical data, the empirical equations do not account for the underlying biology and, thus, are of limited value in simulating the effects of alternate CAR-T designs, cell sources or treatment regimens. A mathematical model capable of quantitatively describing clinical data that is also based on sound biological mechanisms would be useful for the development of novel CAR-T products, as systems pharmacology modeling has proven for other therapeutic modalities^9^.

Mathematical models of T cell–tumor interactions have a long history^7^ and have been adapted to describe various aspects of CAR-T pharmacology, such as antigen binding^8,10^, intercellular signaling^11^ cytokine release^12^, tissue distribution^13^ and competition with host T cells for immune system reconstitution^14^. However, none of the above models adequately defines what limits cell expansion nor what underlies the wide variability in exposure and tumor response observed among patients^15^.

Insights can be gleaned by examining T cell dynamics in response to viral infection. Upon viral antigen encounter, antigen-specific T cells clonally expand and differentiate into cytotoxic effectors, which clear infected cells. After elimination of the pathogen, effector cells undergo a precipitous contraction phase, and a small percentage survive to form long-term memory T cells capable of self-renewal and recall responses. However, if the infection fails to resolve, chronic antigen stimulation leads to T cell exhaustion, wherein remnant T cells lose the ability to produce cytokines, kill target cells or proliferate in response to antigen^16,17^. We hypothesize that an analogous process underlies the pharmacology of CAR-Ts.

We tested this hypothesis using a conceptually simple mathematical model of T cell differentiation control, wherein an antigen-driven toggle switch regulates cell fate transitions among memory, effector and exhausted T cells. We found that the model is capable of quantitatively describing CAR-T pharmacokinetic and tumor dynamic data from multiple clinical trials and deconvolutes biological mechanisms underlying clinical variance. Specifically, we identified cell-intrinsic differences in the proliferation rate of memory cells and cytotoxic potency of effectors as the primary determinants of exposure and response, and we confirmed these mathematical inferences via analysis of bulk and single-cell RNA sequencing (scRNA-seq) data. Population exposure and response predictions were validated against registrational data from Kymriah and Yescarta. Furthermore, we demonstrate that these cell-intrinsic response-mediating differences originate in the CAR-T product using a machine learning workflow that accurately predicts patient outcomes using pre-infusion product transcriptomes. We found that functional gene signatures outperform standard T cell immunophenotyping in predictive accuracy for two CD19-targeted CAR-T products in three indications, and we summarize the relative expression of these signatures across datasets via a CAR-T response scorecard. In summary, the model predicts, de novo, clinical variance in exposure, covariates of response and the biological mechanisms underlying the pharmacology of CAR-Ts.

## Results

### Model structure

**Fig. 1:**
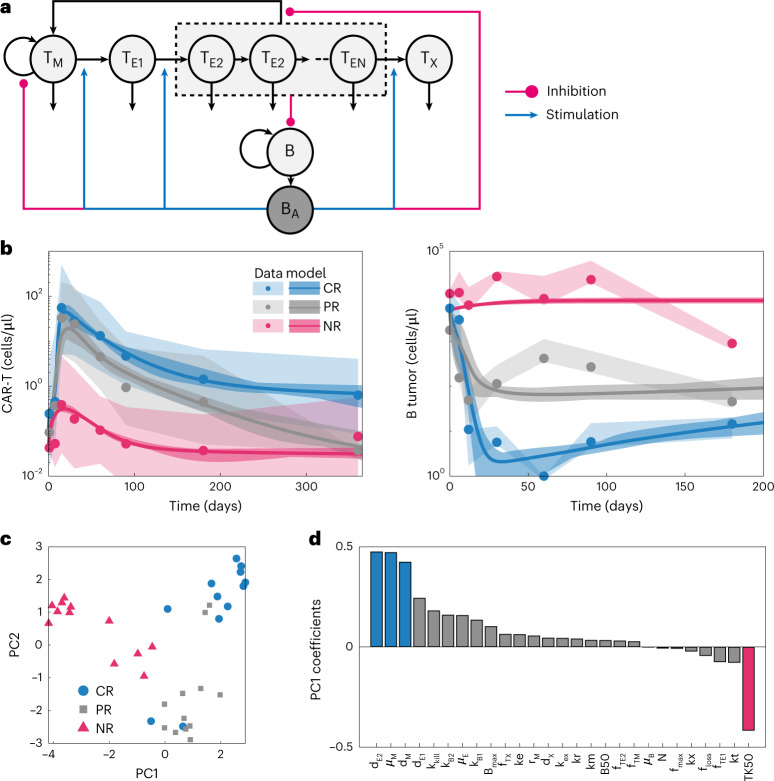
An antigen toggle switch model of T cell regulation quantitatively describes pharmacokinetics/pharmacodynamics behavior of CR, PR and NR patient population response to Kymriah in CLL. **a**, Cartoon depiction of the model structure, comprising three populations of T cells—T memory cells (*T*_*M*_), T effector cells (*T*_*E*1_ and *T*_*E*2_) and exhausted T cells (*T*_*X*_)—and B cell tumors (*B*). Tumor cells express B cell antigen (*B*_*A*_), which stimulates T cell proliferation and differentiation and inhibits the formation of T memory cells. **b**, We fit the model to published pharmacokinetics/pharmacodynamics profiles separated by response category (CR/PR/NR) from Fraietta et al.^18^ using PSO. Model fits (curves: mean of 12 parameter sets; dark shaded areas: middle 90%) agree with both CAR-T and B cell tumor dynamics over time (dots: mean data; light shaded areas: range of data) for each of the three prototypic populations. **c**, PCA plot of the logarithm of the best-fitting parameters colored by population. PC1 captures 35.3% of the variability, and PC2 captures 21.7% of the variability. **d**, Sorted PC1 coefficients suggest that *TK*50 (highlighted pink bar) and *k*_kill_, *μ*_*M*_ and *d*_*M*_ (highlighted blue bars) are the largest sources of variation between CR and NR populations. These parameters correspond to cytotoxic potency, tumor cell lysis rate, memory cell proliferation and death rates, respectively.

We consider T cells (and CAR-T products) to comprise three functionally distinct cell populations: T memory cells (*T*_*M*_), capable of long-term self-renewal and immunological memory; T effectors (*T*_*E*_), responsible for target-mediated cell killing; and exhausted T cells (*T*_*X*_), lacking both killing potential and proliferative capacity. An antigen-sensing toggle switch coordinately regulates the decision of memory cells to self-renew versus differentiate, the rate of effector proliferation, exhaustion and the rate of memory cell regeneration from effectors (Methods). This represents a conceptually simple yet biologically sound description of T cell function and regulatory control in response to immunological need, as determined by systemic antigen burden (Fig. 1a).

### Model parameterization: patients with CLL treated with Kymriah and grouped by response

We first sought to determine whether the mathematical description of T cell regulatory control could quantitatively capture characteristic CAR-T pharmacokinetic and tumor dynamic profiles and whether parameter estimates reveal anything about biological underpinnings of clinical variability. Fraietta et al.^18^ reported mean pharmacokinetic and tumor dynamic profiles of patients with chronic lymphocytic lymphoma (CLL) treated with Kymriah (CTL019, a CD19-targeted CAR-T), grouped by complete responders (CRs), partial responders (PRs) and non-responders (NRs). We digitized the data (mean ± s.d.) and used particle swarm optimization (PSO) to estimate model parameters characterizing the three population archetypes (Fig. 1b). Parameters were estimated 12 times per patient group. Although parameters are non-identifiable (Supplementary Information), the clinical data were captured with good accuracy (Supplementary Fig. 5).

### Biological mechanisms differentiating CR, PR and NR populations

To decipher the biological mechanisms underlying the differing patient response profiles, parameter estimates from the three patient populations were first decomposed into principal components (PCs) (Fig. 1c). Note that the three populations form relatively distinct clusters in parameter space, wherein the *x* axis depicting PC1 (accounting for 35.3% of the variance) separates virtual patients by response, and the *y* axis depicting PC2 (accounting for 21.7% of the variance) separates CR and NR groups from PRs. Examining the coefficients of PC1 (Fig. 1d), the lowest value (associated with NR) is *TK*50 (cytotoxic potency of effectors), and the largest positive contributions (associated with CR) is memory and effector cell turnover (proliferation and death rates; *μ*_*M*_, *d*_*M*_ and *d*_*E*2_). That is, in responding patients, CAR-T effectors lyse target tumor cells much more efficiently, and both memory and effector cells cycle at a higher rate. These findings are consistent with local parameter sensitivity analysis (Supplementary Fig. 6).

It is established that frequency of memory cells in CAR-T infusion products, as assessed by standard T cell immunophenotyping, is predictive of clinical response^19,20^. This was one of the primary conclusions of Fraietta et. al.^18^. However, the PC1 loadings (Fig. 1d) suggest that cell-intrinsic differences in memory cell function (*μ*_*M*_ and *d*_*M*_) rather than frequency (*f*_*Tm*_) are more important determinants of response. To discern the importance of memory cell frequency versus function, we preformed two experiments. First, we attempted to fit the data under the hypothesis that the only difference between CR/PR/NR populations was the composition of the product (frequency of *T*_*M*_, *T*_*E*_ and *T*_*X*_ cells), whereas the cell-intrinsic kinetic parameters are conserved (Supplementary Fig. 7). The model does capture differences in pharmacokinetics and tumor dynamics between the populations, and the inferred CAR-T product composition is consistent with that reported by Fraietta et al.^18^. However, the magnitude of differences between the populations cannot be fully explained by this hypothesis. That is, CAR-T cell composition as defined by memory and exhausted cell frequencies alone is insufficient to explain the variance in clinical activity.

To directly compare the inferred differences in memory cell function among CR/PR/NR groups, we simulated a dose-ranging study using purified memory cell populations from CR/PR/NR archetypes (Supplementary Fig. 8). The CR memory cells produced robust and dose-dependent CAR-T expansion, persistence and tumor reduction, whereas the NR cells showed very little expansion or anti-tumor activity, and the PR memory cells display somewhat intermediate function. In sum, these results imply that, although memory cell frequency in CAR-T infusion products contributes to exposure and response, cell-intrinsic features, such as proliferative capacity, are necessary to account for the variance clinical outcomes. We next sought to identify molecular signatures that underly these cell-intrinsic features and resultant clinical variance.

### Molecular and cellular features differentiating CR, PR and NR populations

To examine the molecular and cellular features underlying these functional differences, we used bulk RNA-seq data from the same trial^18^ wherein pre-infusion CAR-T products were sequenced and annotated by response category. Differential expression analysis on the CR versus NR populations revealed biological features (gene signatures) consistent with inferred functional differences (Supplementary Figs. 9 and 10). We confirmed findings from the original report and additionally found that the CR population is enriched in CD4^+^ and CD8^+^ memory cell gene signatures (defined by single-cell sequencing of thymic tissue^21^) and display heightened expression of signatures characterizing T cell proliferation, effector cytokine (interferon) signaling and IL2RB, IL7 and JAK/STAT signaling (defined by curated pathway databases^22–24^). CAR-T cells from NR patients show heightened p53 (ref. ^25^) and DNA damage^26^ signaling, pathways that may underly the proliferative deficit.

**Fig. 2:**
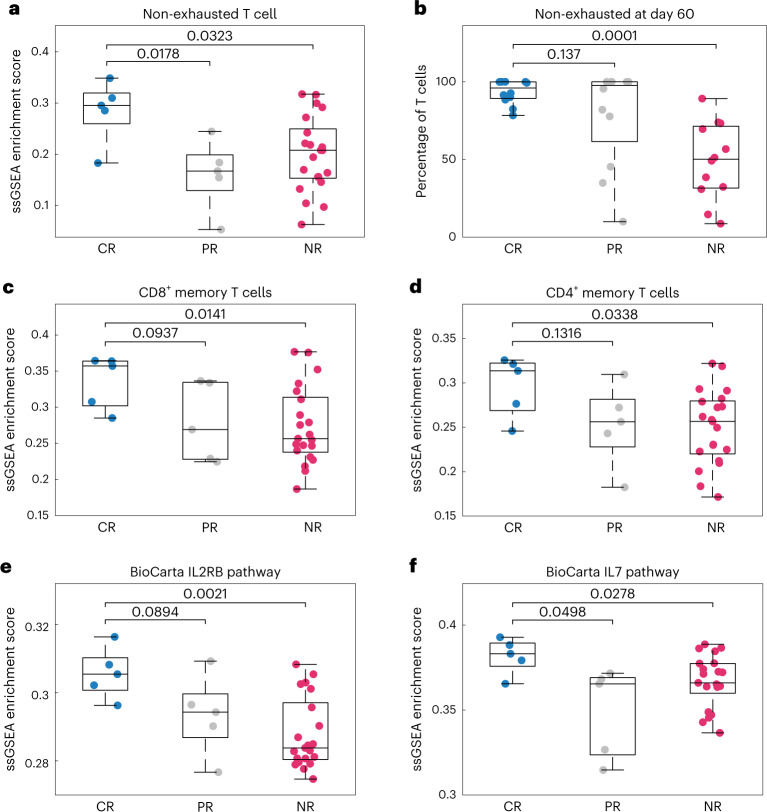
ssGSEA estimates the activity of signaling pathways and enrichment of cell populations in CAR-Ts, separated by response. **a**,**c**–**f**, ssGSEA reveals differences in cell populations and signaling pathways between populations for selected cell signatures and signaling pathways (panel titles). *n* = 31 independent samples—five CR, five PR and 21 NR. **b**, Using the 12 best-fitting parameter sets for each population and model simulations, we calculated the percentage of the T cell population at day 60 that is non-exhausted. The median non-exhausted T cell population at day 60 (over the 12 parameter sets) is near 100% for both CR and PR populations, whereas the median is approximately 50% for the NR population. Differences between populations were assessed using an unequal variances two-sided *t*-test (*P* values shown). Box plots represent median ± 25th percentiles, with whiskers representing min/max values.

Single-sample gene set enrichment analysis (ssGSEA) was subsequently used to examine distribution of the pathway and cell signatures in individual samples. The CR population is significantly enriched in the ‘non-exhausted T cell’ signature (Fig. 2a), consistent with simulations, wherein the fraction of non-exhausted cells at day 60 (peak of anti-tumor effects) is significantly higher in the CR group (Fig. 2b), whereas cells from the NR patients rapidly progress to exhaustion (Supplementary Fig. 11). The simulations also align with clinical reports that CAR-T products that fail to expand in vivo show heightened expression of exhaustion markers LAG3 and PD1 (ref. ^27^).

We found that CRs are differentially enriched in both CD8^+^ and CD4^+^ memory T cell signatures (Fig. 2c,d), consistent with the necessity of memory cells for mediating sustained responses^28^. Note, however, that bulk sequencing data cannot resolve cell population frequencies nor discern between transcriptionally similar versus co-varying cell types (Supplementary Fig. 12). That is, CR products may have higher frequencies of CD4^+^ and CD8^+^ memory cells or may contain cells with more ‘memory-like’ transcriptomes at similar frequencies. The CR population also shows heightened IL2RB and IL7R signaling (Fig. 2e,f), indicating that the CR cell products may show heightened sensitivity to the correspondent cytokines. Notably, IL2 and IL7 are common components of CAR-T expansion media^29^, and peak serum IL7 concentration is predictive of CD19 CAR-T exposure and progression-free survival^30^. Although the results shown in Fig. 2 are statistically significant, the ssGSEA distributions overlap between response categories. Thus, in addition to the limitations of bulk sequencing data, none of the gene signatures assessed could serve as univariable predictors of patient response.

### Cell-intrinsic functional differences mediating CAR-T clinical response

To deconvolute the role of cell frequency versus function in mediating response, we leveraged two recently published clinical studies containing scRNA-seq data of pre-infusion, autologous CD19 CAR-T products matched with clinical outcomes. Bai et al.^31^ reported data for 12 patients with acute lymphoblastic leukemia (ALL) treated with a CD19 CAR-T product analogous to Kymriah—five CRs, two NRs and five patients who relapsed (RL). Haradhvala et al.^32^ reported data for 32 patients with large B cell lymphoma (LBCL) treated with either Kymriah (*n* = 13) or Yescarta (*n* = 19). For the Kymriah-treated group, there were six CRs and seven NRs; for the Yescarta-treated group, there were 11 CRs, one PR and seven NRs.

**Fig. 3:**
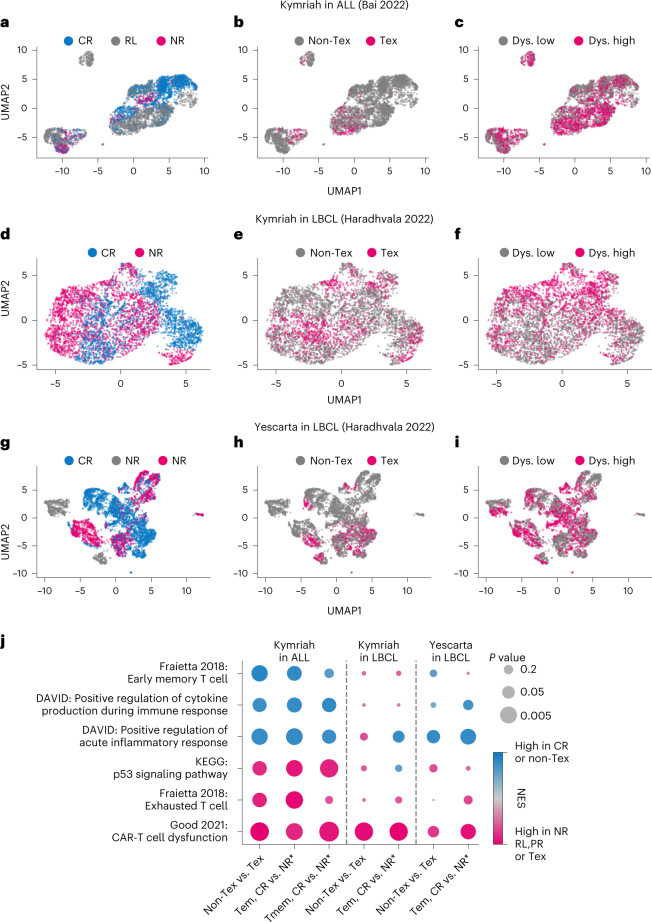
scRNA-seq of pre-infusion CAR-T products reveals cell-intrinsic defects associated with non-durable response. UMAP projections of three datasets representing Kymriah in ALL (**a**–**c**), Kymriah in LBCL (**d**–**f**) and Yescarta in LBCL (**g**–**i**). **a**,**d**,**g**, UMAP projections annotated by response category. **b**,**e**,**h**, UMAP projections annotated as exhausted using ProjectTILs^33^. **c**,**f**,**i**, UMAP projections annotated for high (above mean) or low (below mean) CAR-T cell dysfunction signature from Good et al.^35^. **j**, GSEA for select pathways, comparing both exhausted versus non-exhausted and CR versus PR/RL/NR categories within cells annotated as T effector memory (Tem via ProjecTILs) or early memory (Tmem; CD8^+^CD45RO^−^CD27^+^ via CITE-seq). A positive normalized enrichment score (NES, blue) indicates higher enrichment in CR/non-exhausted cells. *NR = NR/RL or NR/PR. *P* values were calculated by Kolmogorov–Smirnov tests implemented in GSEA.

Examination of uniform manifold approximation and projection (UMAP) projections of the three datasets (Kymriah in ALL, Kymriah in LBCL and Yescarta in LBCL) reveals some separation of response categories in transcriptome space, particularly in ALL (Fig. 3a,d,g). To assess whether response separation is attributable to differences in T cell composition, we assigned cell type labels by mapping expression profiles of the individual cells to annotated tumor-infiltrating lymphocyte populations via ProjecTILs^33^. Most CD8^+^ cells in all three datasets are classified as T effector memory (Tem) or T exhausted (Tex), but there are no consistent differences in composition by response category (Supplementary Fig. 13a–c). For example, the frequency of cells annotated as exhausted is significantly higher in the NR/RL categories as compared to CR in the ALL data (*P* < 0.05, mean 4.4% versus 8.7%, respectively; Fig. 3b,e,h). However, this pattern does not hold for the LBCL data, and the modest effect size is insufficient to account for the vast disparity in clinical outcomes. We used the cellular indexing of transcriptomes and epitopes by sequencing (CITE-seq) antibody tag data provided by Bai et al.^34^ to assign early memory (Tmem: CD8^+^CD45RO^−^CD27^+^) and exhausted (CD8^+^PD1^+^) cell annotations by immunophenotype, reported to be predictive of response in CLL^18^. Although exhausted cell annotations by ProjecTILs and immunophenotype were notably concordant (6.7% versus 5.9% of total cells), cell frequencies did not differ by response category in ALL (Supplementary Fig. 13d,e).

To probe cell-intrinsic function, we annotated cells using a ‘CAR-T dysfunction’ signature, characteristic of functionally exhausted CAR-T cells with reduced proliferative and cytotoxic capacity^35^. Visually, the dysfunction signature is dispersed throughout response categories and not restricted to exhausted regions (Fig. 3g,h,i). Interrogating cell-intrinsic functional differences at a deeper resolution, we performed differential gene expression analysis on T sub-cell populations (annotated both by transcriptome and immunophenotype), followed by pathway enrichment for select gene signatures (Fig. 3j). As a control, we first assessed differences between cells annotated as exhausted versus non-exhausted. Exhausted cells are consistently enriched in the CAR-T dysfunction signature across datasets, whereas the ‘exhausted T cell’ and ‘P53 signaling’ signatures appear specific to the ALL-exhausted cells. Conversely, non-exhausted cells show disparate enrichment for the ‘early memory T cell’ signature as well as cytokine production and inflammatory response signatures, hallmarks of T cell functional potency.

Comparing cell populations from the CR versus NR/PR/RL categories reveals a consistent pattern across datasets. Focusing either on effector memory or early memory (CD8^+^CD45RA^−^CD27^+^) subsets, the NR/PR/RL groups display characteristic features of exhaustion. In particular, the CAR-T dysfunction signature is consistently heightened. The CR cell populations conversely show increased expression of early memory and/or T cell functional signatures (cytokine production and inflammatory response). That is, memory and effector cell populations from CAR-T products resulting in CR appear more functional or ‘memory-like’, whereas the same cell populations from NR/PR/RL categories appear more exhausted. The single-cell data, thus, confirm inferences from the model in separate indications (ALL and LBCL): CAR-T infusion products associated with non-durable response display deficits in proliferative and functional capacity intrinsic to memory and effector cell populations.

### Cell-intrinsic attributes predictive of CAR-T response can be inferred from pre-infusion product transcriptomes

If CAR-T response is product-intrinsic rather than host-intrinsic, we reasoned that the differences in pre-infusion product transcriptomes could be predictive of response. Moreover, comparing response classifiers based on cell-intrinsic function (transcriptome) versus cell composition (T cell phenotype) could help elucidate which product-intrinsic feature is more clinically relevant. We used the bulk RNA-seq data from Fraietta et al.^18^ to develop a multivariate transcriptome classifier. Starting with the 28 pathways that were differentially expressed between the CR versus NR groups (false discovery rate (FDR)-adjusted *P* < 0.05; Supplementary Information), we trained a logistic regression-based classifier using a genetic algorithm for feature selection (Methods).

**Fig. 4:**
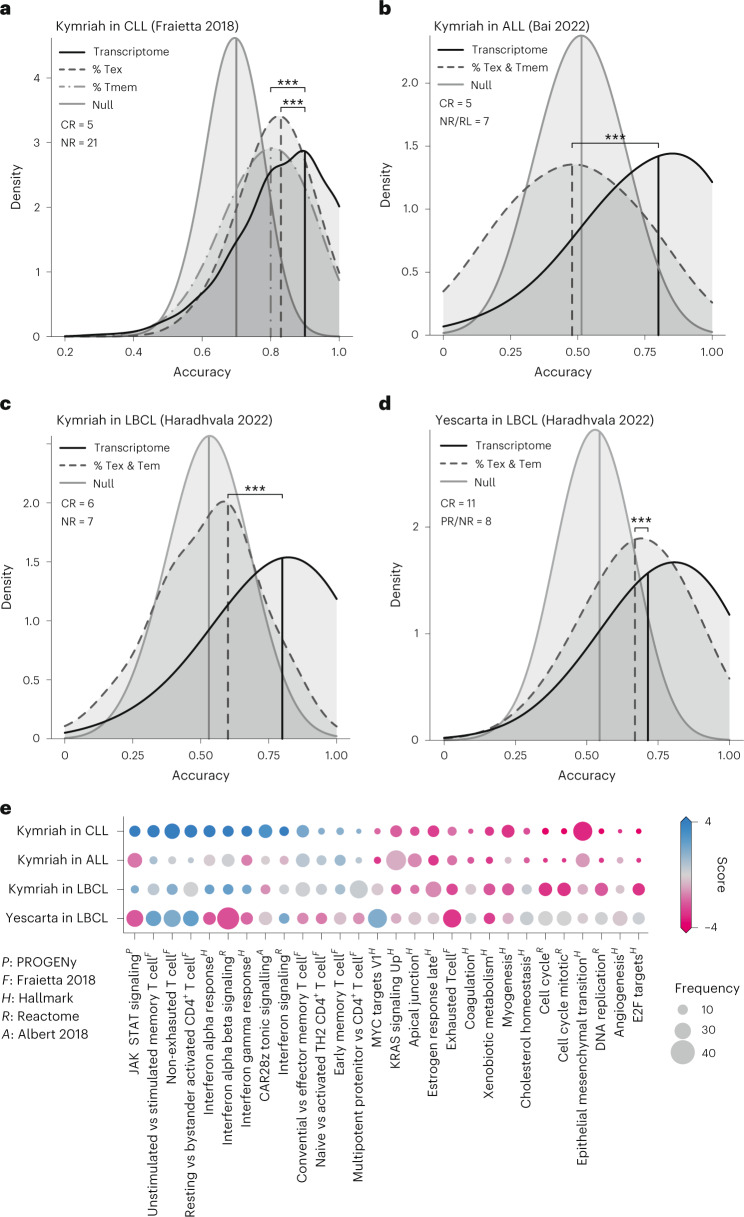
CD19 CAR-T response can be predicted from infusion products using an ssGSEA-based transcriptome classifier with better accuracy than T cell immunophenotypes. Distribution of predictive accuracies are shown for 2,500 iterations using 60:40 train:test split cross-validation. Results from the transcriptome-based ssGSEA classifier are compared to classifiers (**a**) based on reported T memory (CD8^+^CD45RO^−^CD27^+^) and T exhausted (CD8^+^PD1^+^) cell frequencies from Fraietta et al.^18^. **b**, A bivariate classifier based on calculated T memory (CD8^+^CD45RO^−^CD27^+^) and T exhausted (CD8^+^PD1^+^) cell frequencies from Bai et al.^34^. **c**,**d**, Bivariate classifiers based on T effector memory and exhausted cell frequencies from ProjecTILs annotations of Haradhvala et al.^32^. Accuracy distribution resulting from null models (random classification) is shown as controls. *** indicates *P* < 10^−15^, two-sided rank-sum test. **e**, CAR-T response scorecard, representing the 28 gene signatures fed into the transcriptome classifier, ordered by differential GSEA in Fraietta et al.^18^. Bubble size indicates frequency of inclusion in the 2,500 trained models after feature selection; color indicates differential enrichment between response groups by dataset, based on pseudo-bulked GSEA (score = −1 × sign(NES) × log_10_*P* value). Red, CR enriched; blue, NR/PR/RL enriched. Gene signatures are annotated by source. NES, normalized enrichment score.

The resultant model was able to predictively distinguish CAR-T products from CR versus NR patients, with a median cross-validated accuracy of 90% based on a train:test split of 60:40 (Fig. 4a). As comparison, we trained and assessed classifiers using the early memory (CD8^+^CD45RO^−^CD27^+^) and exhausted (CD8^+^PD1^+^LAG3^+^) cell frequencies as reported^18^ (Supplementary Fig. 13d). The resulting accuracies (80% and 83%, respectively) are significantly better than chance but less so than that achieved using functional transcriptomes (*P* < 10^−15^ and *P* = 6 × 10^−11^, respectively). The gene signature panel thus reveals clinical functionality to an extent not apparent from immunotyping, implying that transcriptomes yield more value as CAR-T product characterization assays than current best-practice flow cytometry panels.

To assess whether these findings translated across datasets and indications, we applied the same workflow to pseudo-bulked single-cell data from Bai et al.^34^ (Kymriah in ALL) and Haradhvala et al.^32^ (Kymriah and Yescarta in LBCL). For the Bai et al.^34^ data (Kymriah in ALL), we compared accuracy of classifying CR versus NR/RL groups using the 28-gene signature panel to a bivariate classifier trained using the early memory (CD8^+^CD45RO^−^CD27^+^) and exhausted (CD8^+^PD1^+^) immunophenotype frequencies calculated from CITE-seq antibody tags (Supplementary Fig. 13d). Median accuracy of the transcriptome classifier was 80%, less (as expected) than before but better than that achieved by T cell immunophenotyping (47%, *P* < 10^−15^; Fig. 4b). We similarly assessed predictive accuracy using the LBCL data from Haradhvala et al.^32^ separately for Kymriah and Yescarta. As no immunophenotype data were provided, we compared the transcriptome classifier to bivariate classifiers based on estimated T effector memory (Tem) and exhausted cell (Tex) frequencies from ProjecTILs^33^ annotations (Supplementary Fig. 13b,c). Median predictive accuracy of the transcriptome classifier was 80% and 71% for Kymriah and Yescarta, respectively, outperforming T cell phenotype-based classification in both cases (60% and 67%, *P* < 10^−15^; Fig. 4c,d). As an additional control, we seeded the classifier with ‘random’ pathways by sampling from the compendium of gene signatures that were not differentially expressed between CR versus NR groups in the CLL data (FDR-adjusted *P* > 0.05; Methods and Supplementary Fig. 14). The resulting accuracies were either slightly better or indistinguishable from chance (the ‘null’ model), and all were significantly less accurate than predictions arising from the 28-gene signature panel.

Machine learning models are notoriously difficult to interpret. To condense the inner workings of the transcriptome classifier into interpretable patterns, we created a CAR-T response scorecard (Fig. 4e). This summarizes GSEA on the 28 select pathways and frequency of inclusion in the 2,500 trained models across each of the four datasets. There is variance in the directionality and statistical significance of the signatures between datasets, as would be expected. These represent different diseases, CAR-T products and platforms, and the data were generated by independent groups. However, the overlap is far greater than would be expected by chance (*P* < 10^−5^ for all; Methods). Notably, the Yescarta LBCL scorecard is visually distinct from the three Kymriah scorecards, and the resulting model predictions are correspondingly less accurate. This suggests distinct yet overlapping biology underlying response between the two products.

In summary, response to two separate CD19 CAR-T therapy products (Kymriah and Yescarta) in three indications (CLL, ALL and LBCL) is at least partially predetermined by functional attributes of the CAR-T infusion product. These functional attributes are shared across the four datasets to varying extents, revealed through gene signatures, and not fully apparent from T cell immunophenotyping.

### Explaining inter-patient variability in Kymriah pharmacokinetics

The pharmacokinetics of Kymriah and other CAR-T products tested in clinical trials show high inter-patient variability, with AUCs spanning three orders of magnitude^4,36,37^. Although the transcriptome classifier can predictively distinguish response categories, we assessed whether our mechanism-based model is explanatory of the additional pharmacological variability—specifically, whether a mixture of the three patient archetypes (CR/PR/NR), combined with reported variation in administered dose and initial tumor burden, is sufficient to quantitatively account for the observed variance in exposure.

**Fig. 5:**
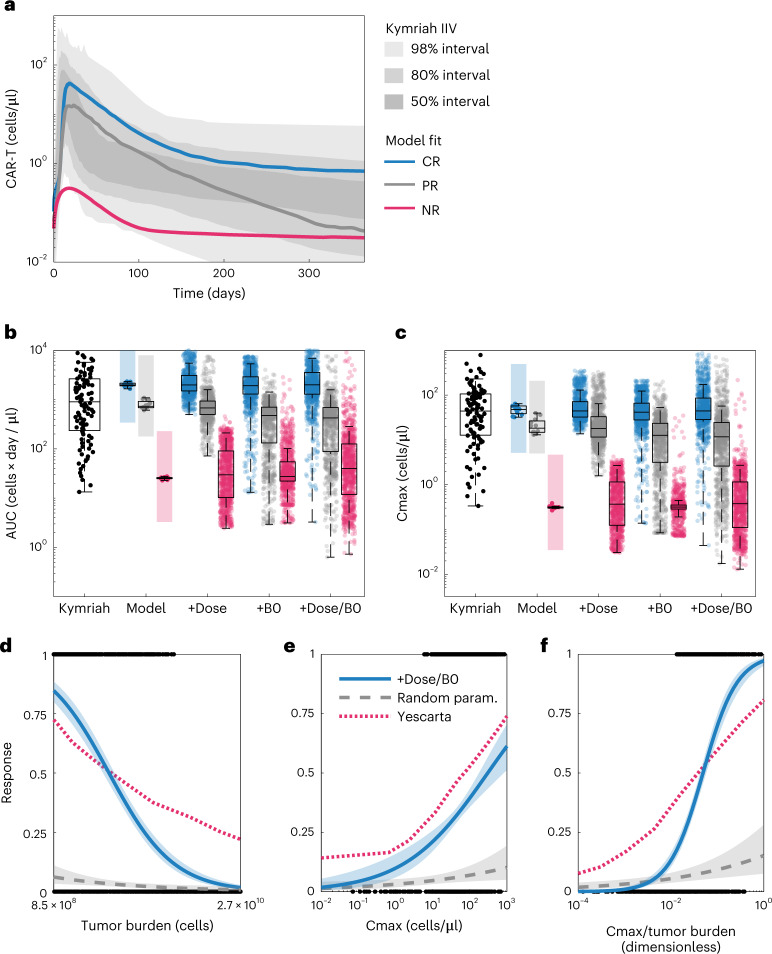
Clinical variability in dose, tumor burden and CR/PR/NR pharmacological archetype account for population variance in Kymriah exposure and predict clinical covariates of response to Yescarta. **a**, Shaded areas show the clinical variability of exposure to Kymriah^5^ with median model simulations overlaid for the CR, PR and NR populations. **b**, CAR-T AUC distributions. The box plot labeled Kymriah shows the distribution in AUC obtained from 1,000 simulations of the clinical pharmacokinetics model (each dot corresponds to a percentile of the AUC distribution). The group of box plots labeled Model shows the AUC distribution obtained for the 12 best-fitting parameter sets for each population (CR, blue; PR, gray; NR, pink) with the colored background the range of AUCs obtained from the clinical pharmacokinetics data. The group of box plots labeled +Dose shows the AUC distributions for each population when doses are randomized within reported ranges in the virtual population (*n* = 1,000); +B0 shows the distributions when initial tumor burdens are randomized; and +Dose/B0 shows the distribution when both dose and initial tumor burdens are randomized. Box plots represent median ±25th percentiles and whiskers the min/max value or an additional 1.5-fold quartile distance. **c**, Cmax distributions plotted as in **a**. **d**–**f**, We defined response to treatment as tumor AUC less than 10,000 cells × day / µl and evaluated whether each patient in the virtual CR population with randomized doses and tumor burdens (+Dose/B0) exhibited a response (black binary data points). Logistic regression with respect to the tumor burden (**d**), Cmax (**e**) or the quotient of Cmax and tumor burden (**f**) reveals how each predicts response (blue curve indicates model estimate with 95% confidence intervals). As a control, uniform random sampling of parameter space (1,000 parameter sets) does not exhibit these response relationships (gray dashed line indicates model estimate with 95% confidence intervals). The clinical covariates of response calculated using the virtual population have the same trends as published covariates of response to Yescarta (red dotted curves). Note that the covariates of response for Yescarta have been linearly scaled to match the ranges in the virtual population for plotting.

We first overlaid simulations of the CR/PR/NR pharmacokinetic profiles with registrational data for Kymriah^5^. Although these are different patient populations (CLL versus B cell ALL (B-ALL)), the pharmacokinetics are highly conserved between these two indications^6^. Visually, the CR/PR/NR profiles correspond roughly to the top quartile, median and bottom 5% of exposure (Fig. 5a). Thus, the CR/PR/NR population archetypes cover much of the pharmacokinetic variation but do not fully account for individual patient variability as they were fit to population means.

We next assessed the effect of variability in dose and tumor burden using a virtual population approach^9^. We created virtual populations (*n* = 1,000) by Monte Carlo sampling across the parameter sets while randomizing dose and tumor burden within reported ranges, either alone or in combination, by log-uniform sampling.

The simulated exposures (AUC) for these virtual populations span the inter-individual variability of Kymriah (10^1^–10^4^ cells × day / μl; Fig. 5b). Variance in either dose or tumor burden is sufficient to cover and roughly match the reported variance of exposure within the CR/PR/NR populations. That is, although the model was fit to population mean data assuming fixed tumor burden and dose, relaxing either of these input assumptions is sufficient to account for reported variance. Similar results are produced by examining the Cmax (Fig. 5c). Grid simulations were used to assess how tumor burden and dose drive exposure and tumor response (Supplementary Fig. 15), revealing a non-linear relationship that likely contributes to the clinical variance. Given that the model recapitulates observed variance in exposure, we next assessed whether these simulations predict clinical covariates of tumor response.

### Predicted covariates of response: Cmax and tumor burden

We examined whether the virtual populations could predict a priori the reported statistical relationships among cell expansion, tumor burden and clinical response. A thorough analysis of response covariates to Yescarta in large cell B cell lymphoma (LCBCL) identified the ratio of CAR-T expansion to initial tumor burden (that is, Cmax/B0) as the strongest correlate of durable response^20^. The same result was reported for overall survival in B-ALL^38^, indicating that this is a conserved feature across indications. The median pharmacokinetics and population variance of Yescarta are similar to Kyrmiah (Supplementary Fig. 16).

Focusing on the virtual CR population, we defined response by the B cell AUC, set to 10^4^ cells × day / µl (the minimum observed for the virtual PR population). We used a logistic regression model linking response to initial tumor burden (B0), Cmax or the ratio as predictors (Fig. 5d–f). The equivalent logistic curves from Yescarta were digitized and overlaid by normalizing the *x* axes. The results are qualitatively consistent with the clinical data, in that these covariates are predictive of response.

To assess whether these predictions emanate directly from the model structure or necessitate model training, we created a ‘control’ virtual population by random sampling of parameter space (*n* = 1,000). This control population did not reproduce the same findings, emphasizing the need for appropriate training data to make accurate predictions.

### Dose–response implications: patients with multiple myeloma treated with Abecma (BCMA-CAR-T)

**Fig. 6:**
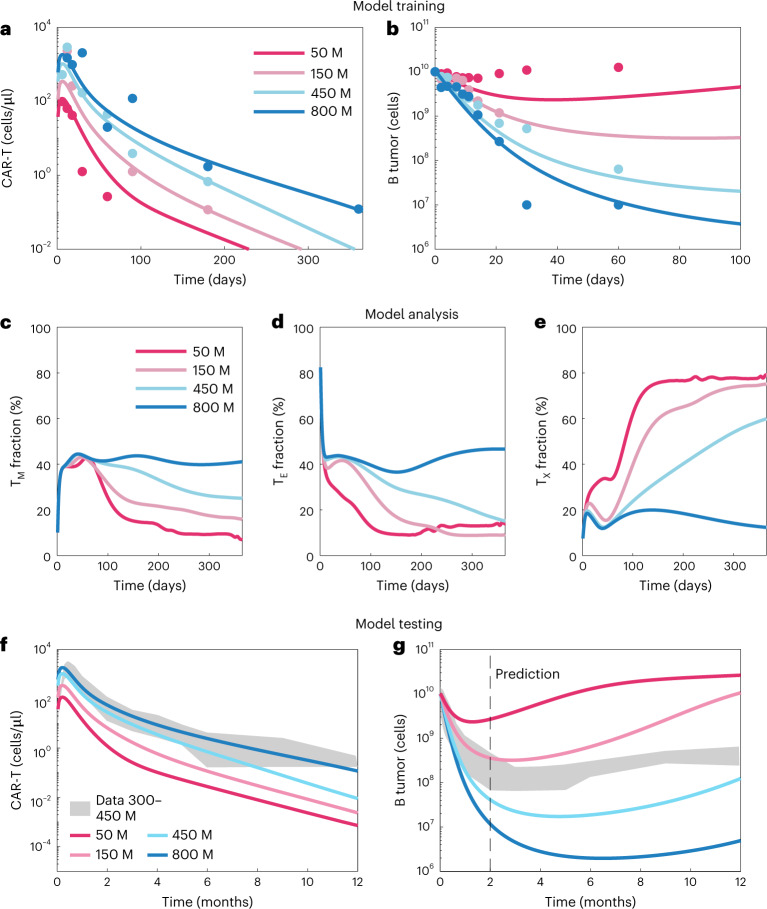
Model extension to Abecma dose response. **a**,**b**, Model training: we fit the toggle switch model to phase 1 dose–response data and observed good fits, with Pearson correlation coefficients from the goodness-of-fit plots (Supplementary Fig. 15) of 0.59 for the CAR-T cells and 0.74 for the tumor. **c**–**e**, Model analysis: we compared the fraction of the total T cell population across doses in the memory, effector and exhausted groups by plotting the mean across parameter sets. For low doses, the T cell population becomes mostly exhausted, whereas, for high doses, the population of memory and effector cells persists. **f**,**g**, Model testing: we compared predictive simulations at two doses with the data reported in the phase 2 study (150–450 million cell doses)^40^. The tumor dynamics out to 1 year fall within the bounds predicted for the 150–450 million cell doses. M, million.

To better understand the relationship among dose, Cmax and tumor response, we applied the modeling framework to a phase 1/2 dose-escalation study of Abecma (BB2121, idecabtagene vicleucel), a BCMA-targeted CAR-T approved for the treatment of multiple myeloma^39^. We again used PSO to estimate model parameters characterizing the pharmacokinetics and tumor dynamics (Fig. 6a,b). Although parameters are non-identifiable, both were captured with good accuracy (Supplementary Fig. 17), and simulations recapitulate the relationship between Cmax/Bo and tumor response identified in Fig. 5f for Kyrmiah and Yescarta (Supplementary Fig. 18).

The simulations yield insight into the effects of CAR-T dose on T cell population dynamics (Fig. 6c–e). The lowest dose (50 million cells) was incapable of tumor reduction and resulted in a predominance of exhausted T cells and gradual loss of memory cells. The highest dose, for which the greatest degree of tumor reduction was observed, produced the opposite response, with minimal exhaustion and a high fraction of memory cells. This is analogous to changes in T cell composition after acute versus chronic infection and provides mechanistic underpinning to the covariates identified above. That is, at an insufficient Cmax:tumor burden ratio, due either to low dose or expansion capacity, the infused CAR-T population will exhaust before clearing tumor.

To assess the predictivity of the model, we compared simulations against data from the phase 2 study, wherein patients were treated at doses of 150, 300 and 450 million cells and tumor dynamics (BCMA levels) were monitored out to 1 year (Fig. 6f,g). Although the pharmacokinetics are moderately under-predicted, the tumor dynamics are predicted with reasonable accuracy. That is, the phase 2 data (150–450 million cell doses) fall between the simulated 150 million and 450 million cell doses with similar dynamics. This is particularly notable, given that the model was trained on data going out to 2 months, whereas predictions are extrapolated out to 1 year.

## Discussion

Multiple clinical studies have confirmed that robust cell expansion after CAR-T infusion is a prerequisite for clinical efficacy^3,20,27,38,40,41^. However, inability to predictively control this pharmacology limits their clinical utility. Mechanism-based mathematical models present a path forward. When trained using appropriate datasets, such models enable the inference of underlying biological principles governing response, enable the ability to generate quantitative predictions and ultimately guide therapeutic design. We hypothesized that the principles governing T cell dynamics during infection also govern the pharmacology of CAR-Ts, and we tested this using a mathematical model of T cell regulatory control, conceptually based on an analogy to a toggle switch. The model was trained using available clinical pharmacokinetic and tumor dynamic data, yielding biological insights and clinical predictions, some of which have been confirmed and some of which remain untested.

First, CAR-T expansion, persistence and anti-tumor response are driven by cell-intrinsic rates of turnover of memory T cell populations and cytotoxic potency of effectors. Using bulk gene expression data, we found that enrichment of memory cell signatures, heightened proliferative and inflammatory signaling and lack of exhaustion markers in pre-infusion CAR-T products correlate with response, consistent with previous work and model-predicted functional differences. Single-cell sequencing data from two additional disease indications and an additional CD19 CAR-T product confirmed that these differences between CR and NR archetypes are intrinsic to memory cell function rather than frequency in the infusion products. CAR-T products resulting in non-durable response show deficits in proliferative and functional capacity characteristic of T cell exhaustion and terminal differentiation, even within immunophenotypically indistinguishable memory and effector cell populations. These functional differences were inferred from the mathematical model and confirmed via expression of a ‘CAR-T dysfunction’ gene signature. We think that CAR-T expansion after infusion (that is, Cmax) represents an in vivo readout of memory T cell proliferative capacity.

We found that response categories can be accurately predicted using pre-infusion product transcriptomes in three indications (CLL, ALL and LBCL) and two CD19-targeted products (Kymriah and Yescarta). Moreover, transcriptome profiles reveal functional attributes not apparent from standard immunophenotyping, and these attributes are shared to varying extents among the datasets examined. Notably, the memory/exhaustion phenotypes identified as predictive of response in CLL did not translate to ALL, whereas the gene signature panel did. Moreover, if pre-infusion product transcriptomes are predictive of response, this implies that these pharmacological archetypes are intrinsic to the infusion product, and, thus, CAR-T efficacy could be improved through product design.

A simple, easily implemented molecular signature for efficacious (CR-like) CAR-T products would be highly valuable for guiding optimization studies. However, such a product-agnostic and indication-agnostic signature remains elusive. Our CAR-T response scorecard reveals transcriptional features that are shared to varying extents among the four datasets. Although there are statistically significant similarities, disparate molecular mechanisms appear to coordinately mediate clinical outcomes among the three datasets and particularly between the two products (Yescarta versus Kymriah). This scorecard could serve as a useful tool for CAR-T product optimization, despite some caveats that are worth noting. First, the pathways selected are derived from the first dataset examined (Kymriah in CLL). It is, thus, a visual representation of the workflow rather than a comprehensive map of features shared consistently across datasets. Second, the colors represent group-level differential pathway enrichment, whereas the classifiers were trained on ssGSEA scores. This compression loses information about the variance within sample groups, which may be important for multivariate classification. The algorithm may, thus, select signatures that do not vary significantly at the group level but nonetheless contain information (that is, large gray bubbles). Finally, many of the signatures make sense biologically (for example, JAK/STAT signaling and exhausted T cell) while others less so (for example, EMT and xenobiotic metabolism). This is an expected outcome of comparing gene lists against pathway databases—many of the signatures are manually curated with inconsistent degrees of validation, and gene lists will overlap between biological processes. We provide the underlying gene sets in the Supplementary Information.

Although efforts are underway to improve CAR constructs and cell culture media, results are constrained by the autologous starting material. Cell-intrinsic differences inferred by the model and highlighted in this scorecard may emanate from the variable ‘quality’ of patient T cells at harvest^42^. If this is the case, reproducible manufacturing of highly efficacious CAR-T products will require a shift from autologous to allogeneic starting material.

We found that pharmacologic archetype, combined with variability in CAR-T cell dose and initial tumor burden, fully accounts for the inter-patient variability in exposure observed in clinical trials of Kymriah. The ratio of CAR-T expansion (Cmax) to initial tumor burden (B0) quantifies whether the cell product infused is capable of clearing tumor, a de novo prediction from the model observed in multiple studies of Yescarta^20,38^. Mechanistically, we predict that cell doses insufficient to clear tumor result in exhaustion of the CAR-Ts, whereas sufficient doses lead to regeneration of memory populations, although no longitudinal phenotyping data are available to assess this.

Controlling the clinical variability in cell dose and initial tumor burden are more immediately tractable problems than optimizing CAR-T cell design. Cell dose has historically been defined by whatever comes out of the manufacturing process, and initial tumor burden as the remnant cancer cells after lymphodepleting chemotherapy, both of which are highly variable among patients. Given consistent quality CAR-T products (for example, those displaying a CR class transcriptional signature), model simulations could be used to define patient-specific doses based on tumor burden (for example, B cell counts) to achieve an optimal balance between maximizing tumor reduction and minimizing Cmax-associated toxicity (Supplementary Fig. 13).

Although our results suggest that the CR versus NR archetype is a product-intrinsic property, delineating product-intrinsic versus host-intrinsic sources of variability is challenging for autologous cell therapies. To start, the definitions are somewhat arbitrary and circular. For our purposes, we define product-intrinsic to mean that clinical response is predictable by properties of the infusion product. These properties (for example, memory cell proliferative capacity) may, in turn, be pre-determined by the patient’s immunological state—a host-intrinsic property. Second, the definitions are blurred as many of the model parameters integrate some aspects of both. Cytotoxic potency (*TK*50), for example, appears to be a cell-intrinsic parameter. However, this lumps together multiple cellular processes: CAR and antigen expression, CAR–antigen binding kinetics, intracellular signal transduction and engagement of cytotoxic machinery. These processes are, in turn, regulated by systemic cytokines and cell–cell interactions. A similar case could be made for most of the model parameters. Thus, although variability in CAR-T dose and tumor burden is sufficient to explain the observed variance in exposure, the inclusion of additional host-intrinsic factors may extend the model’s utility. Tumor-intrinsic signaling^43,44^ and response to lymphodepletion^30^ are two prime examples. Both have been shown to mediate CAR-T expansion and tumor response, as cytokine-mediated interactions among CAR-Ts, host T cells and tumors^14^ likely mediate cell-intrinsic differences.

Additional datasets would be useful to confirm these findings and extend to additional CAR-T products and disease indications. Data availability is, however, limiting. Although hundreds of CAR-T clinical studies have been conducted, raw data from most remain undisclosed, and transcriptome profiling is not routinely implemented. Access to individual patient pharmacokinetics and tumor dynamics profiles, matched with pre-infusion product transcriptomes and well-annotated clinical attributes, would be an ideal starting point to further this work and advance the science.

## Methods

### Clinical data: Kymriah

Mean pharmacokinetic and tumor dynamic profiles were digitized from a clinical study of patients with CLL treated with Kymriah, separated into CRs (*n* = 8), PRs (*n* = 5) and NRs (*n* = 25)^18^. Samples annotated as PR_TD_ (late relapse into B cell lymphoma) were excluded as the profiles are highly similar to the CR patients, and the biological mechanisms underlying such late relapse are unclear. Patients were treated with CAR-T doses ranging from 0.14 × 10^8^ to 11 × 10^8^ cells^41^. For parameter estimation, we assume a fixed dose of 10^8^ cells, consistent with median dose used in this study and other clinical trials of Kymriah. Tumor size data were reported as B cells per microliter and were, hence, used directly in model fitting (assuming an initial tumor burden of 10^10^ total cells). Pharmacokinetics were reported as CD19 CAR transgene copies in peripheral blood (copies per microgram of genomic DNA) and were converted to cell numbers for mechanistic modeling (see below).

The non-linear mixed effects model of Kymriah cellular kinetics, as reported in the BLA^4^ and described in a subsequent publication^5^, was used to simulate population pharmacokinetics in refractory B-ALL. The model was parameterized using data compiled from two clinical studies, treated with a median dose of 10^8^ cells (*n* = 91). Pharmacokinetic profiles of Kymriah in patients with CLL do not to differ substantially from patients with B-ALL^6^. To compute distributions of exposure (AUC and Cmax), we simulated pharmacokinetic profiles for 1,000 virtual patients. At each timestep (0.1 days for 1 year), 1–99 percentiles were computed, and AUC and Cmax were calculated from these percentiles.

### Clinical data: Abecma

Mean pharmacokinetic and tumor dynamic profiles were digitized from a phase 1 dose-escalation study of patients with refractory multiple myeloma (MM) treated with Abecma (*n* = 33), separated by dose group (50, 150, 450 and 800 × 10^6^ cells)^39^. Tumor size data were reported as % change in serum BCMA levels. For model fitting, we assume initial tumor burden as 10^10^ cells and linear scaling between tumor burden and reported soluble BCMA. Pharmacokinetic data were reported as transgene copies per microgram of DNA, and we applied the same scaling factor as above to convert to CAR-T cell counts. Mean pharmacokinetic and tumor dynamic profiles ± s.d. were digitized from a phase 2 study in the same patient population (*n* = 128), treated with 150 × 10^8^ and 450 × 10^6^ cell doses^40^ (data not separated by dose). Tumor dynamic data in this study were reported as serum BCMA (ng ml^−1^). Data were converted to % change from baseline, again assuming initial tumor burden of 10^10^ cells for comparison to model simulations.

### Scaling factors and virtual population

To estimate a scaling factor between transgene counts and cell numbers, we used data from Kalos et al.^45^ wherein both counts per microgram and total circulating CD19^+^ cells were reported, estimated as ~10^4^. For conversions between total cell numbers and cells per microliter for plotting, we assume a total blood volume of 2 L in humans and 2 μl in mice.

### Model structure and assumptions

We encoded three functionally distinct T cell populations: T memory cells (*T*_*M*_), capable of long-term regenerative capacity (self-renewal) and differentiation; T effector cells (*T*_*E*_), which arise from memory population and are responsible for direct killing of tumor cells; and T exhausted cells (*T*_*X*_) that lack effector function and proliferative capacity. T effectors can expand through *N* population doublings but lack the capacity for self-renewal. Alhough the mechanism remains a source of contention, T effectors can regenerate T memory cells after antigen clearance^46^. The core of the mechanism-based description of T cell differentiation control is a toggle switch sensor of tumor antigen, encoded as a Hill equation (a widely used tool in pharmacological modeling^47^). This toggle switch coordinately regulates rates of T memory cell self-renewal versus differentiation, proliferation and exhaustion of T effectors and regeneration of T memory cells from T effectors.

Conceptually, the idea of an antigen sensing, saturable function regulating T cell proliferation was first described by de Boer et al.^48^, extended to differentiation control between memory and effector T cell fates^49^ and applied to CAR-T pharmacokinetics by Martinez-Rubio et al.^50^. A review of published CAR-T pharmacokinetic models in comparison to the below formulation is provided in the Supplementary Information.

We describe this control of T cell fates via a system of non-linear ordinary differential equations:

$$\begin{array}{*{20}{l}} {\frac{{dT_M}}{{dt}}} & = \hfill & {\mu _M \cdot \left(2 \cdot f_{max} \cdot \left( {1 - \frac{{B_A^{km}}}{{B50^{km} + B_A^{km}}}} \right)- 1\right) \cdot T_M + r_M \cdot \left( {1 - \frac{{B_A^{kr}}}{{B50^{kr} + B_A^{kr}}}} \right)}\\&& {\cdot T_{E2} - d_M \cdot T_M,} \\ {\frac{{dT_{E1}}}{{dt}}} & = & {2 \cdot \mu _M \cdot \left( {1 - f_{max} \cdot \left( {1 - \frac{{B_A^{km}}}{{B50^{km} + B_A^{km}}}} \right)} \right) \cdot T_M - \mu _E \cdot \left( {\frac{{B_A^{ke}}}{{B50^{ke} + B_A^{ke}}}} \right)}\\&& {\cdot T_{E1} - d_{E1} \cdot T_{E1}} \\ {\frac{{dT_{E2}}}{{dt}}} & = & {\mu _E \cdot 2^N\left( {\frac{{B_A^{ke}}}{{B50^{ke} + B_A^{ke}}}} \right) \cdot T_{E1} - k_{ex}\left( {\frac{{B_A^{kx}}}{{B50^{kx} + B_A^{kx}}}} \right) \cdot T_{E2} - r_M \cdot \left( {1 - \frac{{B_A^{kr}}}{{B50^{kr} + B_A^{kr}}}} \right)}\\ &&{\cdot T_{E2} - d_{E2} \cdot T_{E2}} \\ {\frac{{dT_X}}{{dt}}} & = & {k_{ex}\left( {\frac{{B_A^{kx}}}{{B50^{kx} + B_A^{kx}}}} \right) \cdot T_{E2} - d_X \cdot T_X} \end{array}$$

Here, the self-renewal and differentiation of memory cells occurs at rate *μ*_*M*_ and is regulated through Hill equation switches that depend on the B cell antigen *B*_*A*_. The parameter *f*_*max*_ describes the fraction of memory cells that self-renew versus differentiate to become effector cells. Memory cells are regenerated (with rate parameter *r*_*M*_) from the *T*_*E*2_ population. We divide the effector populations into two subgroups, *T*_*E*1_ and *T*_*E*2_, that describe the non-tumor killing and tumor killing effector populations, respectively. We made this division for mathematical simplicity: the non-tumor killing subgroup differentiates from the memory cells and forms the initial pool of effector cells that further differentiates (with rate parameter μ_*E*_) to cytotoxic effector cells (*T*_*E*2_). For parameter estimation routines, we encode *N* population doublings in a single source term in the *T*_*E*2_ equation instead of using a hierarchy of ordinary differential equations (ODEs), each tracking the number of cells that have undergone *n* divisions. Because we estimate *N* from data, it would be exceedingly complicated to dynamically update the number of ODEs in the model, as the number of population doublings changes during parameter estimation. T effector cells become exhausted with rate parameter *k*_*ex*_, and all T cell populations are removed with corresponding rate parameters *d*_*M*_, *d*_*E*1_, *d*_*E*2_ and *d*_*X*_. Note that the toggle switch, encoded as a Hill function in B cell antigen *B*_*A*_, has the same half-maximum parameter *B*50 across all T cell populations but different exponents (*km*, *kr*, *km*, *ke* and *kx*) to account for presumed differential dose–response relationships.

We model the dynamics of B cell tumors with logistic growth with rate *μ*_*B*_ and carrying capacity *B*_*max*_ and non-linear tumor killing through effectors with rate *k*_*kill*_, as well as the production and decay of B cell antigen *B*_*A*_:

$$\begin{array}{l}\frac{{dB}}{{dt}} = \mu _B \cdot \left( {1 - \frac{B}{{B_{max}}}} \right) \cdot B - k_{kill} \cdot \left( {\frac{{T_{E2}^{kt}}}{{TK50^{kt} + T_{E2}^{kt}}}} \right) \cdot B\\ \frac{{dB_A}}{{dt}} = k_{B1} \cdot B - k_{B2} \cdot B_A\end{array}$$

By encoding proliferation/differentiation as driven by tumor antigen (*B*_*A*_) rather than simply tumor cell number (*B*), the production degradation rates (*k*_*B1*_ and *k*_*B2*_) create a surrogate transient compartment. This allows for a time delay between changes in tumor burden and responsiveness of T cell fates. Transient compartments are commonly employed in pharmacokinetics/pharmacodynamics modeling^51^ to connect drug concentration to measured pharmacodynamic response.

To map cell dosing to initial condition, we implement two empirical, rapid reactions. First, a proportion of the infused cell dose is rapidly lost to account for discrepancy between cell dose and the initial conditions observed both clinically^45^ and in pre-clinical models^51^ when cells per microliter are reported. Second, the initial cell dose rapidly converts into the four T cell subpopulations. This reaction accounts for the fact that CAR-T products comprise mixed populations of T cells (memory, effector and exhausted states); this composition may vary and is typically not specified in clinical data. Rather than pre-specifying the composition via initial conditions, the rapid conversion reaction allows the fractions to be estimated as model parameters. This is achieved via the following set of equations where *Dose* is the CAR-T dose administered and *DoseX* is the remaining dose that is fractionated into the T cell subpopulations:

$$\begin{array}{*{20}{l}} {\frac{{dDose}}{{dt}}} \hfill & = \hfill & { - \left( {1 + f_{loss}} \right) \cdot Dose,} \hfill \\ {\frac{{dDoseX}}{{dt}}} \hfill & = \hfill & {Dose - \left( {fraction_{TM} + fraction_{TE1} + fraction_{TE2} + fraction_{TX}} \right) \cdot DoseX,} \hfill \\ {\frac{{dT_M}}{{dt}}} \hfill & = \hfill & {fraction_{TM} \cdot DoseX,} \hfill \\ {\frac{{dT_{E1}}}{{dt}}} \hfill & = \hfill & {fraction_{TE1} \cdot DoseX,} \hfill \\ {\frac{{dT_{E2}}}{{dt}}} \hfill & = \hfill & {fraction_{TE2} \cdot DoseX,} \hfill \\ {\frac{{dT_X}}{{dt}}} \hfill & = \hfill & {fraction_{TX} \cdot DoseX.} \hfill \end{array}$$

We applied zero-limits to all cell populations to limit artificial regrowth. That is, if any cell population had a fractional number (<1), that cell population was set to 0. We encoded the model structure in MATLAB SimBiology (R2021a) and used PSO to estimate the model parameters based on minimization of the log mean squared error (MSE) between model simulations and data, using the ‘particleswarm’ function with 100 particles × 100 iterations and the lower limit of quantification (LLQ) set at 10^6^ total cells. We fit the model separately to the CR, PR and NR populations by running the PSO algorithm 12 times for each population, generating a total of 36 parameter sets for analysis (Supplementary Table 1). Model variants based on alternate T cell population structures were also assessed for the ability to fit the data; however, none outperformed the above formulation (Supplementary Information and Supplementary Figs. 1 and 2). To assess generalizability of the model, we also fit to two pre-clinical datasets with pharmacokinetic and tumor dynamic dose–response data: CD19-CAR-T-treated NALM xenografts^52^ (Supplementary Fig. 3) and BCMA-CAR-T-treated MM1.S xenografts^51^ (Supplementary Fig. 4). In both cases, the model described the data with good accuracy. See Table 1 for a list of model parameters, units and lower and upper bounds used in the PSO algorithm.

**Table 1:** Model parameters, units and PSO bounds

Parameter	Description	Units	Lower bound	Upper bound
*B*50	Antigen toggle switch half-maximum	Number of antigen molecules	10^6^	10^10^
*μ*_*B*_	B cell proliferation rate	1/day	0.001	0.1
*k*_*kill*_	Rate of B cell killing by T effectors	1/day	0.001	1
*f*_*loss*_	Fraction of dose lost	1/day	1	1000
*TK*50	B cell killing half-maximum	Cells	10^5^	10^9^
*kt*	B cell killing Hill exponent	Dimensionless	0.2	3
*k*_*B*1_	Antigen generation rate	Number of antigen/(day×cell)	0.001	1
*k*_*B*2_	Antigen clearance rate	1/day	0.001	1
*μ*_*M*_	T memory proliferation rate	1/day	0.001	1
*km*	T memory self-renewal Hill exponent	Dimensionless	0.2	3
*f*_*max*_	T memory maximum fraction of self-renewal	Dimensionless	0.5	0.99
*ke*	T effector proliferation Hill exponent	Dimensionless	0.2	3
μ_*E*_	T effector proliferation rate	1/day	0.001	1
*N*	Number of population doublings in *T*_*E*2_	Dimensionless	4	12
*k*_*ex*_	T effector exhaustion rate	1/day	0.001	1
*d*_*M*_	T memory death rate	1/day	0.001	1
*d*_*E*1_	T effector (*T*_*E*1_) death rate	1/day	0.001	1
*d*_*E*2_	T effector (*T*_*E*2_) death rate	1/day	0.001	1
*d*_*X*_	T exhausted death rate	1/day	0.001	1
*B*_*max*_	B cell tumor carrying capacity	Cells	10^8^	10^12^
*kx*	T exhaustion Hill exponent	Dimensionless	0.2	3
*kr*	T memory regeneration Hill exponent	Dimensionless	0.2	3
*r*_*M*_	T memory regeneration from T effectors	1/day	0.001	1
*fraction*_*TM*_	T memory fraction of dose	1/day	1	10
*fraction*_*TE*1_	T effector (*T*_*E*1_) fraction of dose	1/day	1	10
*fraction*_*TE*2_	T effector (*T*_*E*2_) fraction of dose	1/day	30	70
*fraction*_*TX*_	T exhausted fraction of dose	1/day	5	30

### Local parameter sensitivity analysis

Local parameter sensitivity coefficients (LPSCs) were computed by simulating the model and computing the CAR-T AUC and tumor AUC in response to a 10% increase in estimated parameter values across the 36 parameter sets characterizing CR/PR/NR populations. We calculated coefficients based on the median change in AUC for each population according to the formula:

$$LPSC_{Y|X} = \frac{{\Delta Y/Y}}{{\Delta X/X}} \cdot 100$$

wherein *Y* is the specified model output (CAR-T or tumor AUC), and *X* is the specified parameter.

### Virtual populations

Virtual populations were created from the CR/PR/NR population fits by Monte Carlo sampling underlying parameter sets while varying CAR-T dose (10^7^–10^9^ cells) and initial tumor burden (8.5 × 10^8^–2.7 × 10^10^ cells) within reported ranges by log-uniform sampling.

### Modeling workflow

Our strategy for model-based integration of the disparate datasets was to (1) fit the pharmacokinetics/pharmacodynamics model independently to the Fraietta et al.^18^ CR, PR and NR profiles; (2) create virtual populations from this model and compare the predicted population pharmacokinetic variance against Kymriah data from Stein et al.^5^ and covariates of response against Yescarta data from Locke et al.^20^; and (3) fit the pharmacokinetics/pharmacodynamics model to Abecma dose–response data from Raje et al.^39^ to understand mechanisms underlying the response covariates.

### RNA-seq analysis

Analysis of bulk RNA-seq data was implemented within R version 4.1.1. In brief, read count data were downloaded from the supplement provided by Fraietta et al.^18^. Trimmed mean of M-values (TMM) normalization was implemented with edgeR (3.34.1), and normalized data were converted to log(counts per million) by applying Voom transformation. Differential gene expression analysis was implemented with limma (3.50.3)^53^ and gene signature analysis with ssGSEA^54^. Normalized ssGSEA scores were calculated as:

$${{N}}ij = \frac{{{{A}}ij - {{{\mathrm{MIN}}}}\left( {{A}} \right)}}{{{{{\mathrm{MAX}}}}\left( A \right) - {\mathrm{MIN}}\left( A \right)}}$$

wherein *A* is the matrix of ssGSEA signature scores (*i*) × samples (*j*). Gene signatures for cell signaling pathways were compiled from PROGENy^25^ (10), BioCarta^22^ (217), Reactome^24^ (674), Hallmark^23^ (50) and DAVID^26^ (6,577). Cell population signatures were derived from those published in Fraietta et al.^18^ (7), a single-cell atlas of thymic development^21^ (13) and individual signatures for CAR-T dysfunction^35^ and CD28z tonic signaling^55^ and are provided in the Supplementary Information.

### scRNA-seq and CITE-seq analysis

scRNA-seq counts and associated metadata for Bai et al.^31^ and Haradhvala et al.^32^ were retrieved from the Gene Expression Omnibus (GSE197215 and GSE197268, respectively). Gene counts were normalized using Seurat (4.1.0), and cell type labels were assigned using ProjecTILs^33^ (2.2.0) with the default scRNA-seq-based reference atlas of tumor-infiltrating lymphocytes. Differential expression analysis was implemented with Seurat using a Wilcoxon rank-sum test, followed by GSEA. ssGSEA scores were calculated using GSVA (1.40.1) and used without normalization as input features to the classifier. For CITE-seq-based immunophenotyping, we called each cell as positive/negative based on reference to the associated control antibody tag.

### ssGSEA-based response classifier

ssGSEA scores corresponding to all gene signatures that were differentially enriched between CR and NR groups in Fraietta et al.^18^ (28, based on an FDR-adjusted *P* < 0.05) were used to build a logistic regression-based classifier of response status:

$$log\left( {\frac{{p\left( {CR} \right)}}{{1 - p\left( {CR} \right)}}} \right) = \beta _0 + \beta _1 \cdot ssGSEA_1 + \beta _2 \cdot ssGSEA_2 + \ldots + \beta _N \cdot ssGSEA_N$$

wherein p(CR) is the probability of complete response (versus non-response), and *β*_*i*_ are regression coefficients. A genetic algorithm, implemented in R with the glmulti package (1.0.8), was used for feature selection on the 60% training split of the data, using the Akaike information criterion (AIC) with model accuracy as the objective function. Model accuracy is defined as:

$$Accuracy = \left( {TP + TN} \right)/\left( {TP + TN + FP + FN} \right)$$

wherein TP, TN, FP and FN refer to true positive, true negative, false positive and false negative. For the genetic algorithm, we used a population size of 100 with a mutation rate of 0.001, an immigration rate of 0.3 and a reproduction rate of 0.1. Owing to the stochastic nature of genetic algorithms, this was repeated 2,500 times, wherein each iteration produced a list of *N* pathways to be used as features for logistic regression. For randomized control models, we randomly selected 2≤*N*≤6 pathways from the remnant pathway compendium (7,520, FDR-adjusted *P* > 0.05) as input features, using an *N* distribution based on observed frequencies in the trained models. Predictive accuracy was assessed using the 40% test split of the data and model accuracy distributions compared via Wilcoxon rank-sum tests and visualized as kernel density estimates with manually chosen bandwidths. Immunophenotype classifiers were developed using the same workflow excluding feature selection, with input features being either reported cell frequencies from Fraietta et al.^18^ computed cell frequencies from Bai et al. CITE-seq data^34^ or computed cell frequencies from ProjecTILs^33^ annotation of Haradhvala et al.^32^ data.

Binomial tests were used to assess GSEA overlap in CR versus NR/PR/RL comparisons among datasets. Starting with the top 28 gene signatures identified as differentially expressed in Fraietta et al.^18^ and used to seed the transcriptome classifier, 13/28, 13/28 and 15/28 are significant at a level of *P* < 0.05 in the Bai et al.^34^ and Haradhvala et al.^32^ Kyrmiah and Yescarta datasets, respectively. Of the 7,548 signatures in our compendium, 1,123, 742 and 751 met this level of significance, corresponding to *P* values of 6 × 10^−5^, 7 × 10^−7^ and 10^−8^.

### Software

Model simulations and analysis were performed using MATLAB R2021a and the SimBiology toolbox (6.1). All bioinformatics analysis was done on Ubuntu 20.04.3 LTS running R 4.1.1 (‘Kick Things’). Key packages were GSVA (1.40.1) for ssGSEA, fgsea (1.21.2) for GSEA, celldex (1.2.0) for obtaining reference datasets for SingleR (1.6.1), Seurat (4.1.0), data.table (1.14.2), limma (3.50.3), edgeR (3.34.1), Matrix (1.4.3) and ggplot2 (3.3.6) for data wrangling and visualization.

### Reporting summary

Further information on research design is available in the Nature Portfolio Reporting Summary linked to this article.

## Online content

Any methods, additional references, Nature Portfolio reporting summaries, source data, extended data, supplementary information, acknowledgements, peer review information; details of author contributions and competing interests; and statements of data and code availability are available at 10.1038/s41587-023-01687-x.

### Supplementary information

Supplementary Information Supplementary text and figures. A review of published CAR-T mathematical models, model structural analyses and Supplementary Figs. 1–18.

Reporting Summary

Supplementary Data 1 Parameter matrices for CR, PR, NR and Abecma model variants.

Supplementary Data 2 Cell population gene sets derived from literature.

Supplementary Data 3 Gene sets for 28 signatures used in the classifier.

Supplementary Data 4 Gene sets and enrichment statistics for CR versus NR/PR/RL group comparisons in the Fraietta et al., Bai et al. and Haradhvala et al. Kymriah and Yescarta datasets.

Supplementary Data 5 Twenty-eight pathways used as input features to the classifier, with frequencies of inclusion.

## Data Availability

Single-cell RNA sequencing counts and associated metadata for Bai et al.^31^ and Haradhvala et al.^32^ were retrieved from the Gene Expression Omnibus (GSE197215 and GSE197268, respectively). Bulk RNA sequencing and associated metadata from Fraietta et al.^18^ were downloaded from the supplement, and all additional data were digitized from published figures using Graph Grabber version 2 (Quintessa).

## Code availability

The MATLAB and R code used in this study is provided for non-commercial use via a Zenodo repository: 10.5281/zenodo.6886414 (ref. ^56^).
