# Author Correction: Deconvolution of clinical variance in CAR-T cell pharmacology and response

**DOI:** 10.1038/s41587-023-01816-6

**Published:** 2023-05-15

**Authors:** Daniel C. Kirouac, Cole Zmurchok, Avisek Deyati, Jordan Sicherman, Chris Bond, Peter W. Zandstra

**Affiliations:** 1 Notch Therapeutics, Vancouver, BC Canada; 2 School of Biomedical Engineering and Michael Smith Laboratories, University of British Columbia, Vancouver, BC Canada

**Keywords:** Computational models, Data integration, Predictive markers, Transcriptomics, Predictive medicine

Correction to: *Nature Biotechnology* 10.1038/s41587-023-01687-x. Published online 27 February 2023.

In the version of this article initially published, there were conversion errors in the display equations presented in the “Model structure and assumptions” section of the Methods and the “Model structural assessment” section of the Supplementary Information. The errors have been corrected in the HTML and PDF versions of the article.

