# Supplementary material for: Deconvolution of clinical variance in CAR-T cell pharmacology and response
Source: Nat Biotechnol. 2023 Feb 27;41(11):1606–17. doi: 10.1038/s41587-023-01687-x (PMC10635825; doi:10.1038/s41587-023-01687-x)
Supplement: Supplementary file 1 — Supplementary text and figures. A review of published CAR-T mathematical models, model structural analyses and Supplementary Figs. 1–18. [file 41587_2023_1687_MOESM1_ESM.pdf]

nature biotechnology

Article

https://doi.org/10.1038/s41587-023-01687-x

Deconvolution of clinical variance in CAR-T cell pharmacology and response

In the format provided by the authors and unedited

Supplemental Information

Review of published CAR-T pharmacokinetic/pharmacodynamic models

Chaudry et al.[1] have described a taxonomy of CAR-T cell kinetic models, proposing a few requisite properties:

1. Capture the typical 3 phase pharmacokinetic profiles – exponential expansion, rapid decline, then long term decay.
2. CAR-T expansion is driven by antigen – multiple clinical and pre-clinical observations note that expansion (Cmax) correlates with initial tumor burden.
3. CAR-T cell expansion is limited – insufficient cell doses are incapable of clearing the tumor.
4. Absence of limit cycles – many models predict oscillations of CAR-T and tumor cell numbers, which have not been observed clinically nor in pre-clinical models.

The 'gold-standard' pharmacokinetic model published by Stein et al.[2] and now used by the FDA is purely empirical. It describes CAR-T pharmacokinetics (property 1), but does not include tumor dynamics, thus falls short of properties 2 and 3.

Published mechanism-based models are all derivatives of the predator-prey formulation[3], wherein tumor (antigen) stimulates T cell proliferation, and T cells in turn kill tumor cells, satisfying property 2 by design. However, in all published variants, T cells are endowed with unlimited proliferative capacity; limited only by the loss of tumor (the growth of T cells follows a mass-action law dependent on T cells and tumor cells). As such, a single infused CAR-T cell, given time to expand, will eventually clear tumor [4–6], thereby missing property 3. Predator-prey models are also prone to limit cycles (e.g., Martinez-Rubio et al.[4]). Oscillations are theoretically possible in many feedback-regulated dynamical systems, and clinically manifest in rare cyclic hematological disorders [7]. However, oscillations in either circulating CAR-T or tumor cell counts have not been reported to our knowledge in any pre-clinical or clinical data, though these could be missed due to sampling limitations and patient variability.

Two approaches have been taken to enable predator-prey model formulations to address properties 2 and 3. Singh et al. [8,9] implement a predator-prey model, but encode CAR-T proliferation using a Hill-equation driven by CAR:Antigen complexes per tumor cell, such that low CAR-T doses deliver insufficient receptor to breach this threshold [8,9]. The dose-response simulations thereby display a switch-type behavior with respect to both CAR-T exposure and tumor response: cell doses below a given threshold result in minimal CAR-T expansion or tumor response, and those above result in complete response, with time-to-Cmax being dose-responsive. While this (partially) addresses requisite properties 3 and 4, it suggests that complete responses can be achieved by simply dosing above the threshold and does not explain the observed clinical variability. Moreover, it necessitates an estimate of CAR expression on T cells and antigen expression on tumor tissue, as well as in vitro data to parameterize the relationship between CAR:antigen formation, T cell proliferation and cytotoxicity.

The model published by Kimmel et al. [10] is predator-prey based, but also implements competition between host T cells and CAR-Ts for limited 'space', thereby limiting CAR-T expansion. In this model the *a priori* differentiating factor between patients with robust CAR-T expansion (and tumor clearance) vs. poor CART expansion (and tumor progression) is the kinetics of host immune system reconstitution. This hypothesis is supported by clinical data on the role of lymphodepletion in mediating CAR-T expansion and efficacy [11]. However, it does not preclude the involvement of alternate, product-intrinsic mechanisms in limiting cell expansion and underlying patient-to-patient variability.

Our model formulation makes a few fundamental changes to the typical predator-prey structure to address the four requisite properties and incorporate fundamental T cell biology. Borrowing from the stem cell field, we encode each T memory (T_M) cell division as a fate choice between self-renewal and differentiation, driven by tumor antigen (B_A). CAR-T differentiation and expansion thus occur at the expanse of depleting the pool of memory cells. Effector cells (T_E) cannot self-renew, but rather undergo a fixed number (N) of divisions. This is a novel feature absent from other mechanism-based CAR-T models and addresses the unlimited CAR-T expansion capacity embedded in predator-prey models. Accounting of memory cell self-renewal vs. differentiation also provides a mechanism by which chronic antigen stimulation (or alternatively, insufficient CAR-T dose relative to tumor size) drives exhaustion. If tumor cells cannot be cleared sufficiently to reduce systemic antigen burden below a defined threshold ($B50$), T_M cells will continually differentiate until the pool of long-term memory cells is depleted.

We have also included an exhausted T cell state, notably absent from all above CAR-T models. We believe this is necessary to capture the divergence between CAR-T pharmacokinetics and cytotoxic function, particularly in partial and non-responding patients (explored in detail below).

Model structural assessment

To systematically assess the model topology, we created a series of variants with alternate T cell population structures:

1. T_E population only (effector state)
2. T_M and T_E (memory and effector states), no T_X
3. T_E and T_X (effector and exhausted states)
4. T_M, T_E, and T_X states, but without effector to memory differentiation ($r_M = 0$)
5. Inclusion of additional naïve (T_N) state to the original model.

We illustrate these model structures as cartoons in **Figure S1**.

Note the original (complete model) describes 4 sub-populations regulated by antigen exposure via the ODEs:

$$\frac{dT_M}{dt} = \mu_M \cdot \left(2 \cdot f_{max} \cdot \left(1 - \frac{{B_A}^{km}}{B50^{km} + {B_A}^{km}}\right) - 1\right) \cdot T_M + r_M \cdot \left(1 - \frac{{B_A}^{kr}}{B50^{kr} + {B_A}^{kr}}\right) \cdot T_{E2} - d_M \cdot T_M,$$

$$\frac{dT_{E1}}{dt} = 2 \cdot \mu_M \cdot \left(1 - f_{max} \cdot \left(1 - \frac{{B_A}^{km}}{B50^{km} + {B_A}^{km}}\right)\right) \cdot T_M - \mu_E \cdot \left(\frac{{B_A}^{ke}}{B50^{ke} + {B_A}^{ke}}\right) \cdot T_{E1} - d_{E1} \cdot T_{E1}$$

$$\frac{dT_{E2}}{dt} = \mu_E \cdot 2^N \left(\frac{{B_A}^{km}}{B50^{km} + {B_A}^{km}}\right) \cdot T_{E1} - k_{ex} \left(\frac{{B_A}^{kx}}{B50^{kx} + {B_A}^{kx}}\right) \cdot T_{E2} - r_M \cdot \left(1 - \frac{{B_A}^{kr}}{B50^{kr} + {B_A}^{kr}}\right) \cdot T_{E2} - d_{E2} \cdot T_{E2}$$

$$\frac{dT_X}{dt} = k_{ex} \left(\frac{{B_A}^{kx}}{B50^{kx} + {B_A}^{kx}}\right) \cdot T_{E2} - d_X \cdot T_X.$$

For model **variant 1**, we describe the single effector compartment, wherein proliferation/self-renewal is driven by antigen:

$$\frac{dT_{E2}}{dt} = \mu_E \left(\frac{{B_A}^{km}}{B50^{km} + {B_A}^{km}} \right) \cdot T_{E2} - d_{E2} \cdot T_{E2},$$

For model **variant 2**, we employ the full model, but set $k_{ex} = 0$ such that no exhausted T cells are generated.

For model **variant 3**, we employ a version of variant 1, wherein effectors both proliferate/self-renew and transit to exhausted cells in an antigen-dependent manner.

$$\frac{dT_{E2}}{dt} = \mu_E \left(\frac{{B_A}^{km}}{B50^{km} + {B_A}^{km}} \right) \cdot T_{E2} - k_{ex} \left(\frac{{B_A}^{kx}}{B50^{kx} + {B_A}^{kx}} \right) \cdot T_{E2} - d_{E2} \cdot T_{E2}$$

$$\frac{dT_X}{dt} = k_{ex} \left(\frac{{B_A}^{kx}}{B50^{kx} + {B_A}^{kx}} \right) \cdot T_{E2} - d_X \cdot T_X.$$

For model **variant 4**, we employ the original set of model equations, but set $r_M = 0$ such that memory cells cannot arise from effectors. Note the origin of long-term memory cells remains a point of contention among immunologists. It is established that following clearance of infection, antigen-specific effectors are replaced by antigen-specific memory cells. These were previously assumed to arise via de-differentiation from a subset of differentiation effectors [12] (as coded in our model), though new evidence suggests that these arise from a rare population of stem-like cells [13]. There is data supporting both lineage models [14,15] , and differing sub-sets of memory cells may follow both paths [16,17]. This variant thus assesses whether this teleological de-differentiation reaction is necessary within our model framework, given the limited number of T cell states considered for parsimony.

For model **variant 5**, we have included a naïve T cell compartment (T_N) preceding the memory compartment, as per canonical T cell differentiation hierarchy [12]. These cells proliferate and differentiate to memory T_M cells in an antigen-dependent manner, via the equation:

$$\frac{dT_N}{dt} = 2 \cdot \mu_N \cdot f_N \cdot \left(1 - \frac{{B_A}^{kn}}{B50^{kn} + {B_A}^{kn}} \right) \cdot T_N - d_N \cdot T_N$$

T_N cells differentiate into the memory cell compartment, such that the T_M balance equation is now:

$$\frac{dT_M}{dt} = \mu_M \cdot \left(2 \cdot f_{max} \cdot \left(1 - \frac{{B_A}^{km}}{B50^{km} + {B_A}^{km}} \right) - 1 \right) \cdot T_M + 2 \cdot \mu_M \cdot f_{max} \cdot \left(1 - \frac{{B_A}^{km}}{B50^{km} + {B_A}^{km}} \right) \cdot T_M + r_M \cdot \left(1 - \frac{{B_A}^{kr}}{B50^{kr} + {B_A}^{kr}} \right) \cdot T_{E2} - d_M \cdot T_M$$

This introduces five additional free parameters into the model:

μ_N: the naïve T cell proliferation rate
f_N: the naïve T cell probability of self-renewal
k_N: the Hill exponent linking antigen exposure to naïve T cell proliferation
d_N: the naïve T cell death rate
$fraction\_T_N$: the fraction of CAR-T cell dose in the naïve T cell compartment

The resulting model fits to the CR/PR/NR populations from Fraietta et al.[18] for the five structural variants as compared to the full model are shown in **Figure S2**. Note that all model variants are essentially equivalent with respect to their ability to describe the tumor dynamics but differ substantially in the ability to capture the CAR-T pharmacokinetics.

Model selection for non-linear dynamical models is inherently challenging given the lack of appropriate quantitative metrics. The Akaike Information Criterion (AIC) is widely used, ranking models based on fitting error (MSE) vs. complexity (number of free parameters): [19]:

$$AIC = n \cdot \ln[MSE] + \frac{2 \cdot k \cdot n}{n-k-1}$$

Wherein *n* = number of measurements, *k* = free parameters and MSE = mean squared error.

However, this was originally developed to rank multivariate linear regression models rather than non-linear ODEs and prioritizes limiting free parameters over goodness-of-fit. More fundamentally, MSE essentially considers all data points to be of equivalent value, while subjectively we know some datapoints and readouts are more-or-less significant. Given the tumor dynamic profiles look quite similar across variants, we reasoned tumor fits are not important model selection criterion. The most clinically relevant criteria is efficacy, and CAR-T exposure metrics (Cmax and AUC) are known to predict clinical efficacy. We thus evaluated all model variants by MSE of the Cmax (log10-cells) and AUC (log10-cells.day) in addition to the MSE of all the data and the sample-size corrected AIC:

Variant	Parameters (k)	MSE (all data)	AICc (all data)	MSE (CART Cmax)	MSE (CART AUC)
0	27	0.148	56.94	**0.023914**	**701.89**
1	12	1.477	51.31	0.238173	2633451.7
2	23	0.194	**24.82**	0.12383	1777.4
3	18	1.398	77.38	0.283941	1969201.3
4	26	0.241	66.01	0.458429	2322.9
5	32	**0.052**	140.4	0.059707	850.18

**bold indicates top ranked model by metric*

Based on MSE of all the data, variant 5 (inclusion of the T_N cell compartment) is the most accurate, outperforming the original model (variant 0). Examination of the PK curves in **Figure S1** reveals this improvement is due to capturing the last time point (12 month) of the NR profile, which increases from the previous (6 month). We believe this may be an artefact of the data (population average) rather than a real phenomenon, implying the model is overfitting. Note that model variant 5 contains five additional parameters as compared to the original model, and the resulting AIC more than doubles from 57 to 140, indicating this additional complexity adds little value.

Based on the AICc, variant 2 (lacking an exhausted state but containing T_M and T_E,and the reversable transitions) is ranked highest. However, this version does not adequately capture the pharmacokinetics of the NR population, which reads out as higher MSE of the CAR-T exposure metrics (Cmax and AUC). The deficiency of using AICc as a model selection metric is apparent by examining the curves for model variant 1 (lacking T_M and T_X compartments). While this is ranked higher than the original model due to the reduced number of free parameters, the PK curves do not even closely resemble the data.

Using the exposure metrics (both Cmax and AUC), the original model (variant 0) best captures the data. Consideration of the fitting error, model complexity, and assessment of exposures, we feel the original model outperforms all structural variants.

Note that selection of non-linear models is somewhat subjective. We view mathematical models as caricatures of biological systems; quantitative tools that should capture dynamic organizing principles and explain the data in broad strokes. A model's value lies in what we can learn from it, rather than how

complex and detailed it is. That is, the different variants assessed (and perhaps others not considered) could be equally valuable. These fitting metrics help decide which models to learn from, but do not necessarily imply one as true and others false.

Supplemental Figures

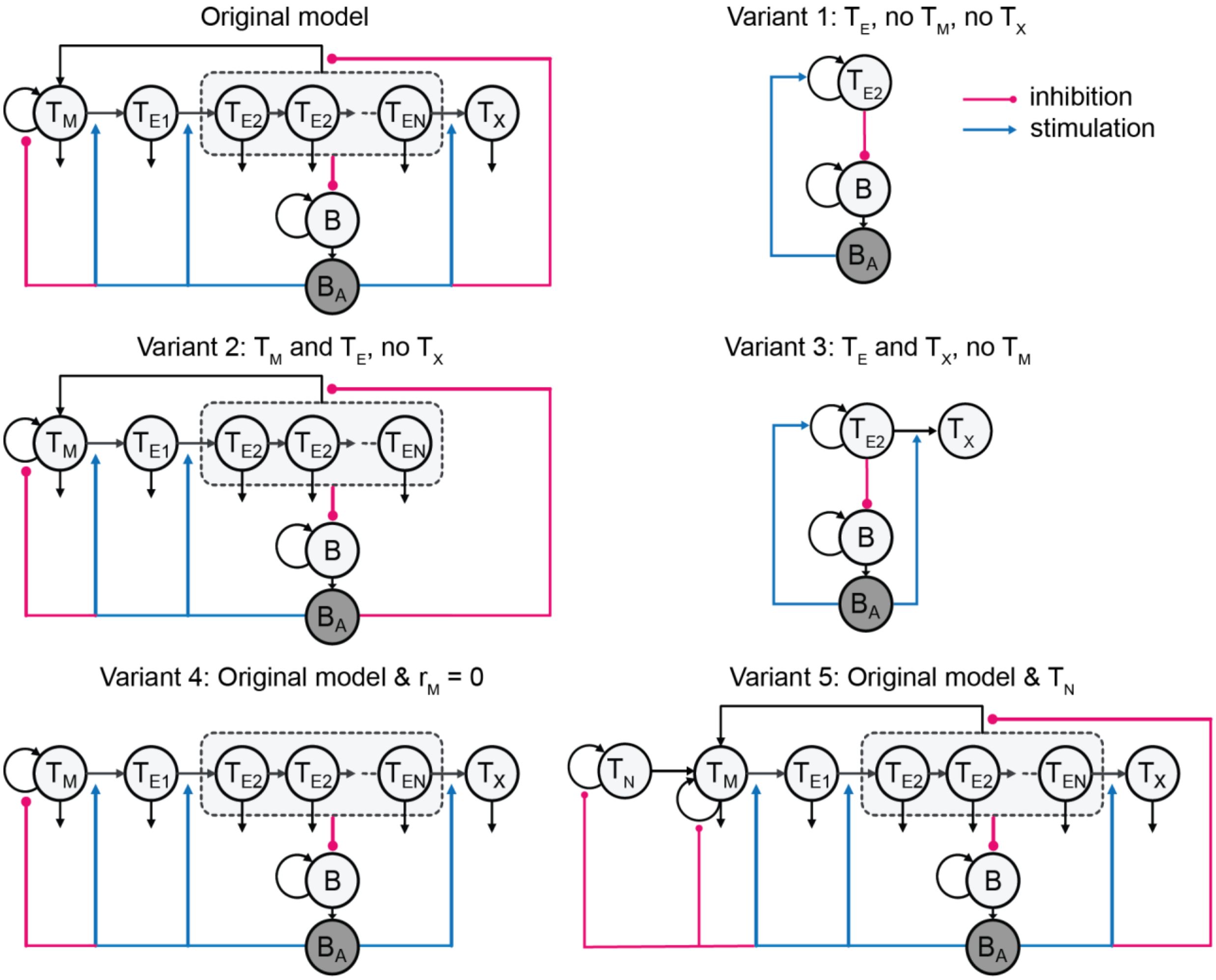

Figure S1: Structures of original model and variants.

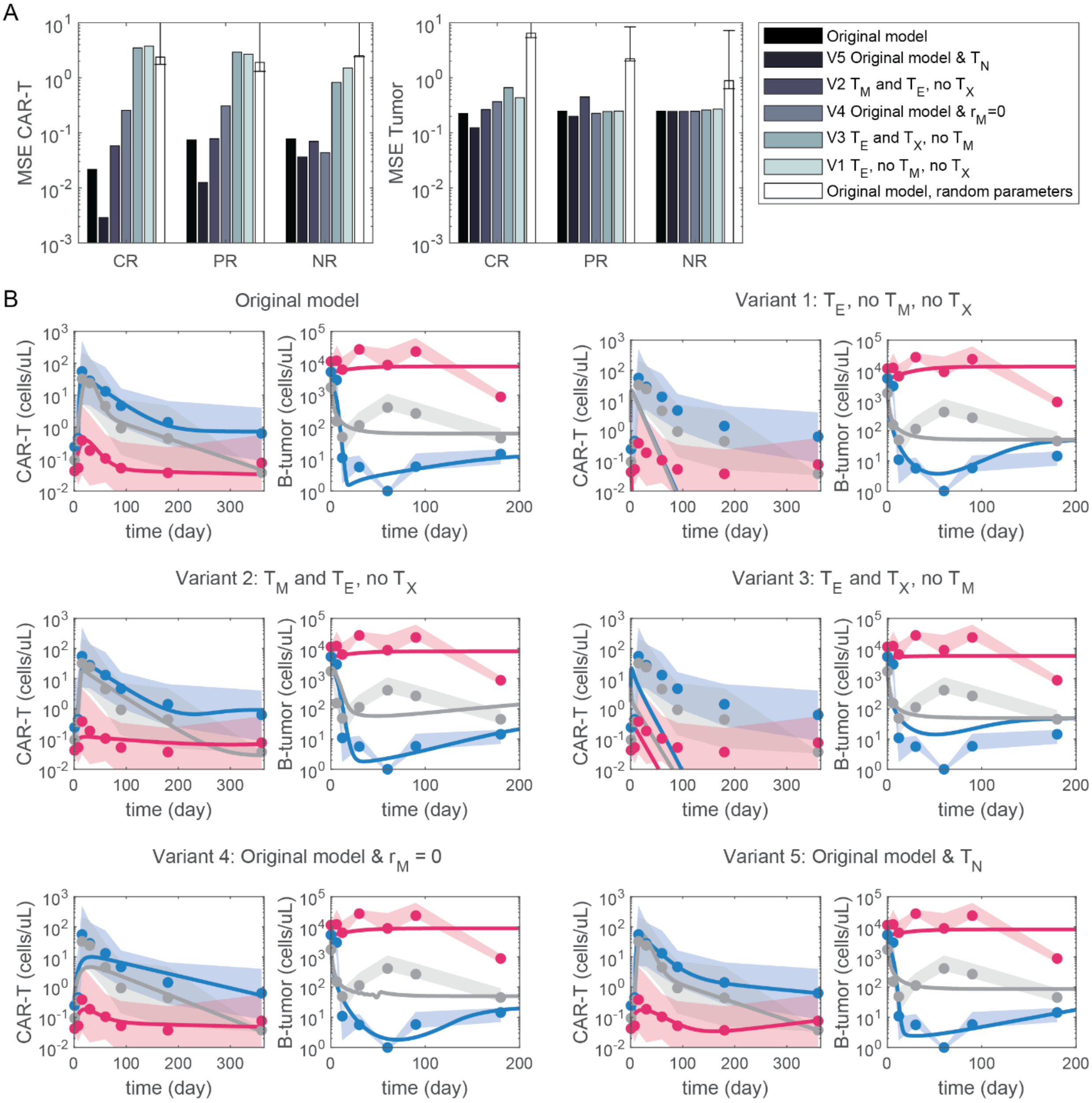

Figure S2: Fitting accuracy of the original proposed model against five structural variants. (A) Mean squared error (MSE) of the original (full) model and the five model variants fit the training data [18], as well as random sampling of parameter search space for the original model (n=100, error bars = std). MSE plots are separated by fit to the pharmacokinetic and tumor dynamics, and rank ordered by overall goodness of fit. (B) Model simulations overlaid with training data for the original (full) model and five variants.

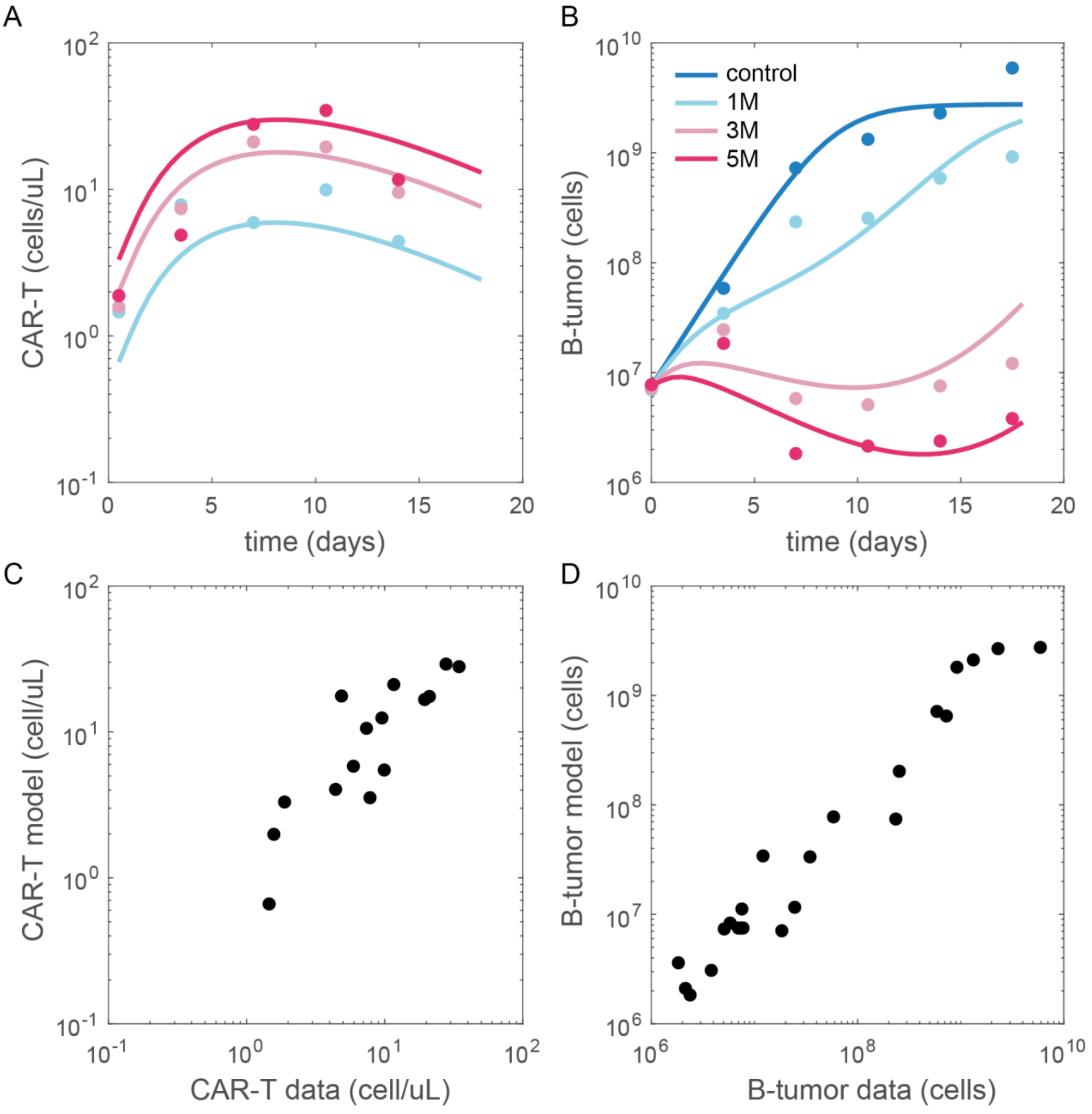

Figure S3. Model fitting to pre-clinical CD19-CAR-T data. NALM-6 xenograft bearing mice (injected with 106 tumor cells at day -6) were treated with increasing doses of Kymriah, and tumor size measured by fluorescence imaging [20]. We assume for simplicity a 1:1 scaling relationship between photons/s and tumor cell number. For fitting mouse as compared to clinical data, we scaled down bounds on the tumor-related parameters Bmax (maximum tumor size) and TK50 (T cell EC50 driving tumor cell killing) and allowed the tumor growth rate (uB) to float between 0.1 and 1 per day. (A) Pharmacokinetic and (B) tumor dynamic data and model simulations for CAR-T doses of 0, 1, 3 and 5 million cells. Goodness of fit plots for the CAR-T pharmacokinetic (C) and tumor dynamics (D), with Pearson correlation coefficients of 0.88 and 0.84 respectively.

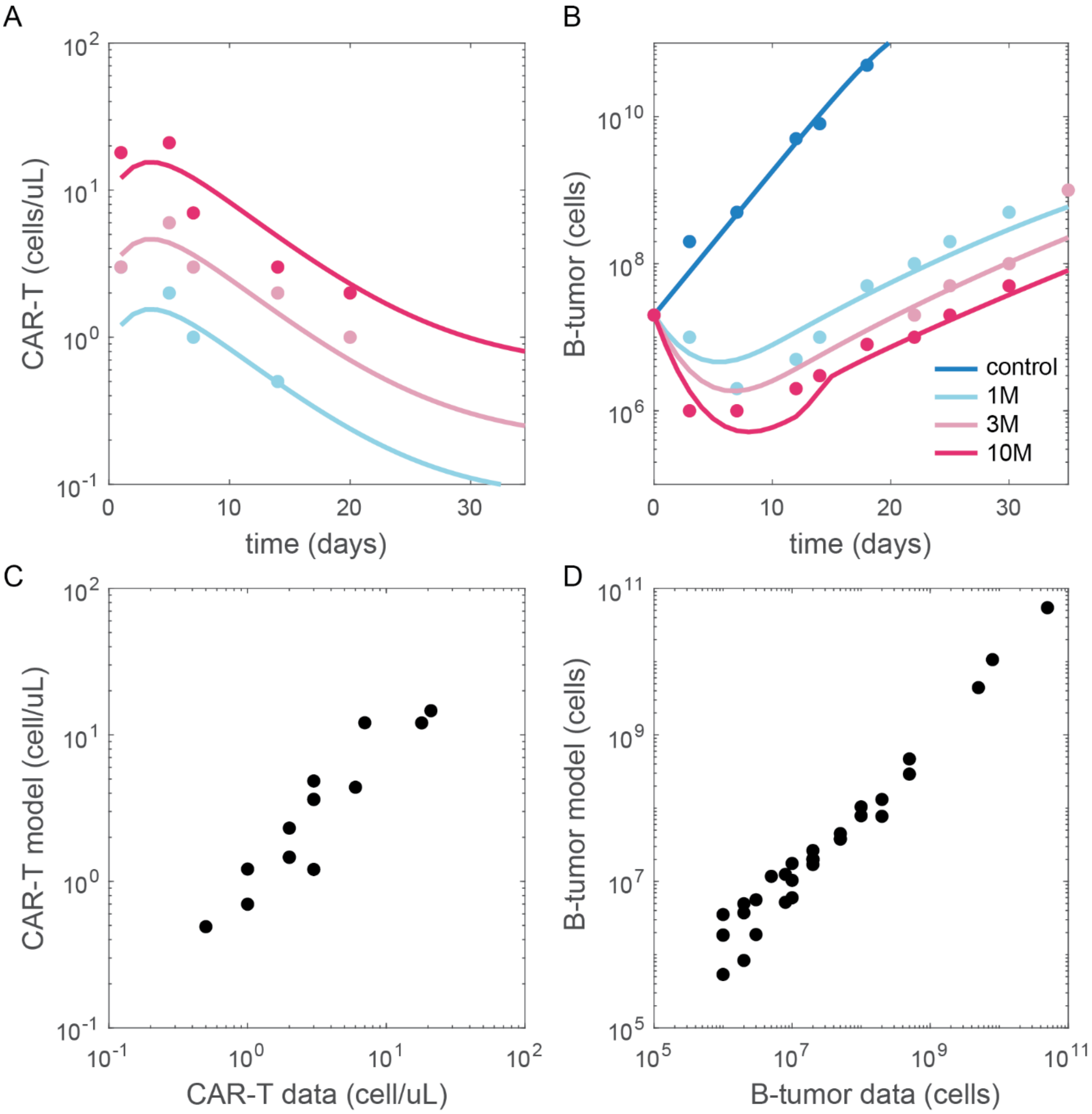

Figure S4. Model fitting to pre-clinical BCMA-CAR-T data. MM1.s xenograft bearing mice (injected with 5x106 tumor cells at day -14 to -8) were treated with increasing doses of the research-grade CAR-T 'BCMA-R2', and tumor size measured by fluorescence imaging [21]. We assume for simplicity a 1:1 scaling relationship between photons/s and tumor cell number. For fitting mouse as compared to clinical data, we scaled down bounds on the tumor-related parameters Bmax (maximum tumor size) and TK50 (T cell EC50 driving tumor cell killing) and allowed the tumor growth rate (uB) to float between 0.1 and 1 per day. (A) Pharmacokinetic and (B) tumor dynamic data and model simulations for CAR-T doses of 0, 1, 3 and 10 million cells. Goodness of fit plots for the CAR-T pharmacokinetic (C) and tumor dynamics (D), with Pearson correlation coefficients of 0.92 and 0.99 respectively.

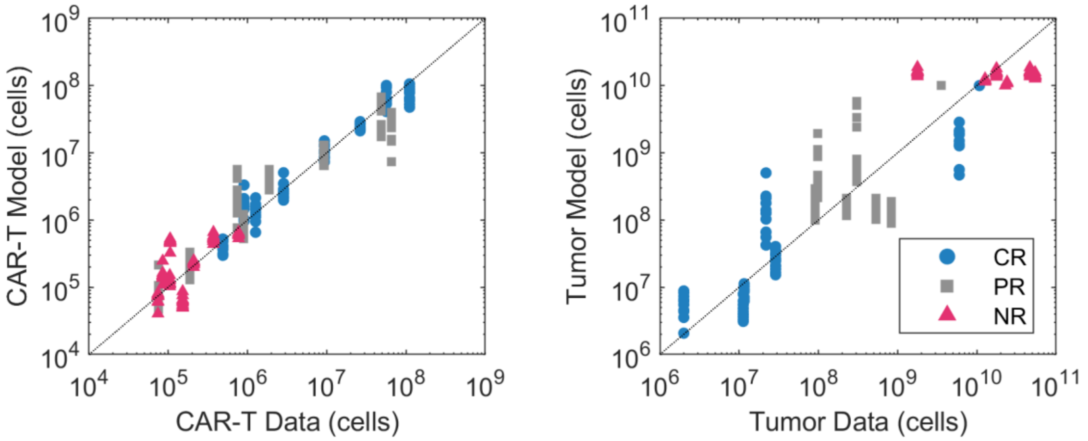

Figure S5: Goodness-of-fit plots for Kymriah model fitting in Figure 1. Accuracy was quantified by computing Pearson correlations of the goodness-of-fits (log-simulations vs. log-data), separated by CAR-T and tumor kinetics for the CR, PR, and NR populations. For the CAR-T pharmacokinetics, median correlation coefficients for the CR, PR, and NR populations are 0.89, 0.88, and 0.76, respectively. The model therefore captures the majority of variance in the PK data for all three groups. For tumor dynamics, the correlations are 0.78, 0.77, and -0.23, respectively. Note the tumor growth portion of the model is very minimal (logistic equation) and captures the overall trends and differences between populations while missing aspects of the dynamics. The CR tumor kinetics are captured relatively well. For the PR and NR groups, the model describes the dynamics of initial tumor size reduction (or lack thereof), but is not the subsequent dynamics. Both the PR and NR tumor dynamics appear to fluctuate up to day-100, then decline, though these may represent sampling artifacts rather than real dynamic features.

We further quantified accuracy by compared the mean squared errors resulting from the set of estimated model parameters to that obtained by random sampling of parameter search space (n=100). *P*-values for the three groups were all $< 10^{-7}$ (rank-sum test), indicating the optimized parameters represent a small segment of parameter space.

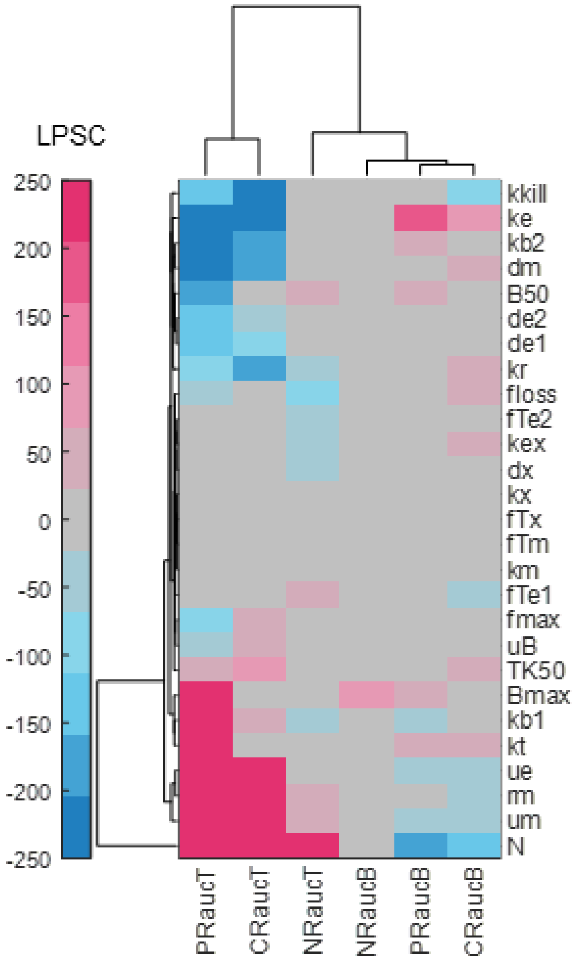

Figure S6: Local Parameter Sensitivity Analysis of CR/PR/NR populations. Local Parameter Sensitivity Coefficients (LPSC) were calculated for CR, PR, and NR populations using the AUC of T cells (aucT) and AUC of B cells (aucB) as outputs, with samples and outputs organized by agglomerative hierarchical clustering. Memory cell proliferation (μ_M) was identified as a critical driver of exposure and response in the CR and PR populations. TK50 was not a locally sensitive parameter despite being identified in the PCA as an important parameter (Figure 1D). The rate of tumor cell lysis (k_{kill}) was the most sensitive parameter mediating tumor response in the CR population. The rate of memory cell regeneration (r_M) and the number of effector cell doublings (N) were additionally found to mediate both CAR-T exposure and tumor response in CR and PR populations, despite not varying significantly between the groups.

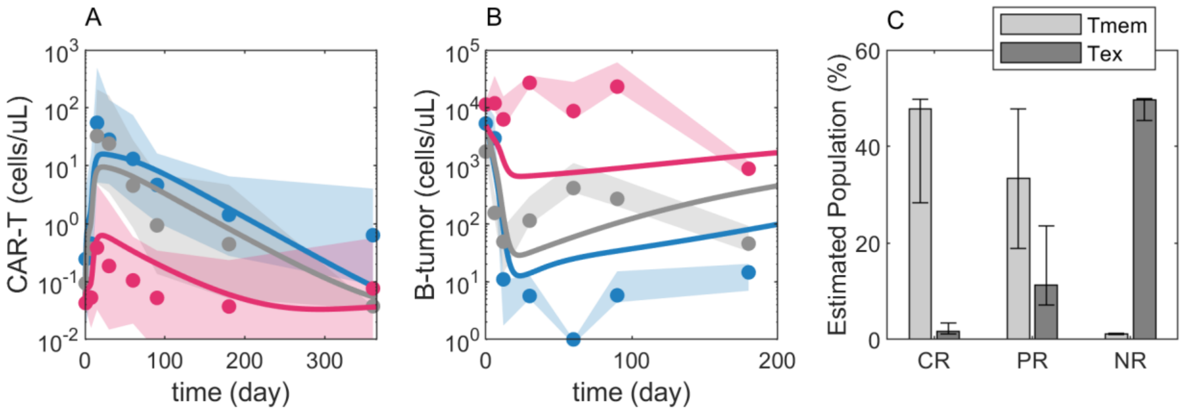

Figure S7. Model fitting results based on the hypothesis that the only distinguishing feature between CR, PR, and NR populations is the fraction of memory T and exhausted T cells in the CAR-T infusion product. Memory cell fraction (*f_Tm*) and exhausted cell fraction (*f_Tx*) were estimated as between 1-50% independently for the CR, PR, and NR populations while all other model parameters were estimated simultaneously using a single vector for the CR, PR, and NR populations. Simulations of best fit model (estimated by MSE minimization) from 12 optimization runs for (A) CAR-T pharmacokinetics and (B) tumor dynamics. (C) Estimated fraction of memory and exhausted cells (*f_Tm*, *f_Tx*) in CAR-T infusion products for CR, PR, and NR populations. Bars represent medians ± 25 percentile intervals from n=12 model fits.

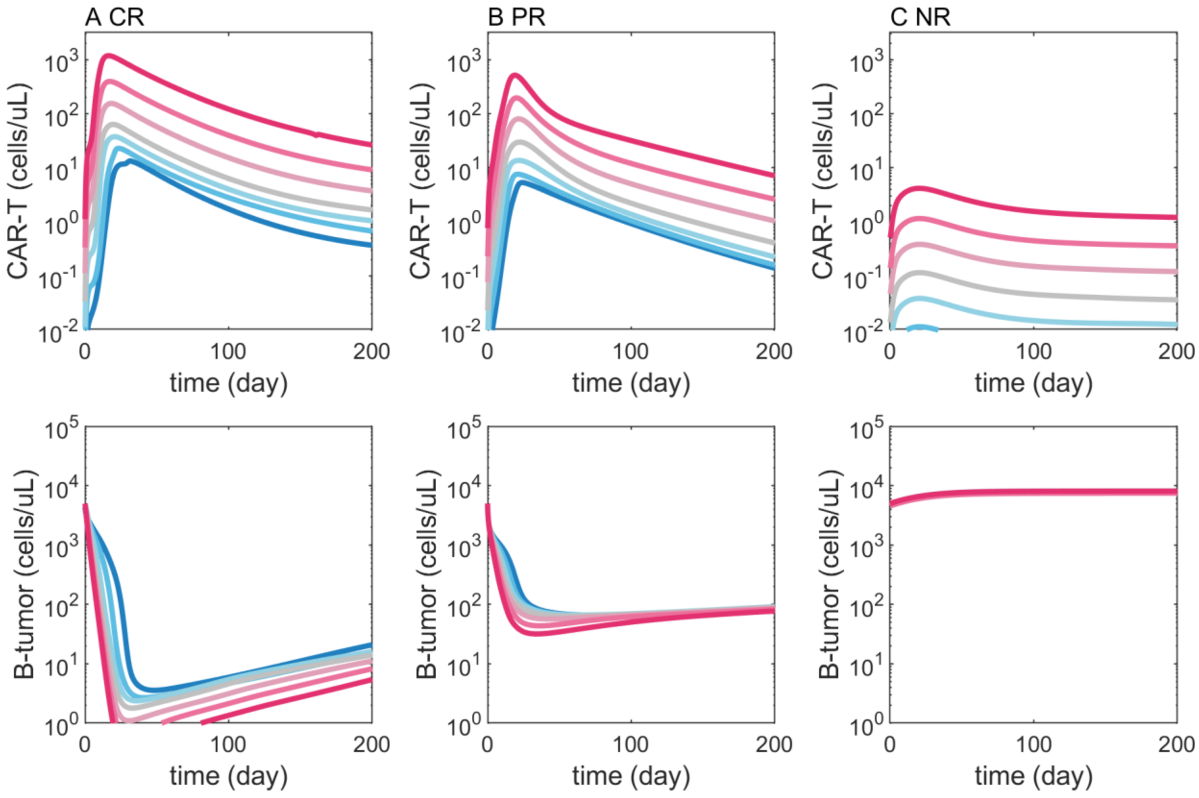

Fig S8. Simulated pharmacokinetic and tumor dynamic responses to increasing cell doses of pure memory cell populations from CR, PR, and NR population models. Simulations were run at doses of 1, 3, 10, 30, 100, 300 and 1000 million cells using parameter sets estimated for CR (A), PR (B), and NR (C) populations. For direct comparison, the memory cell fraction was set to 100% for each.

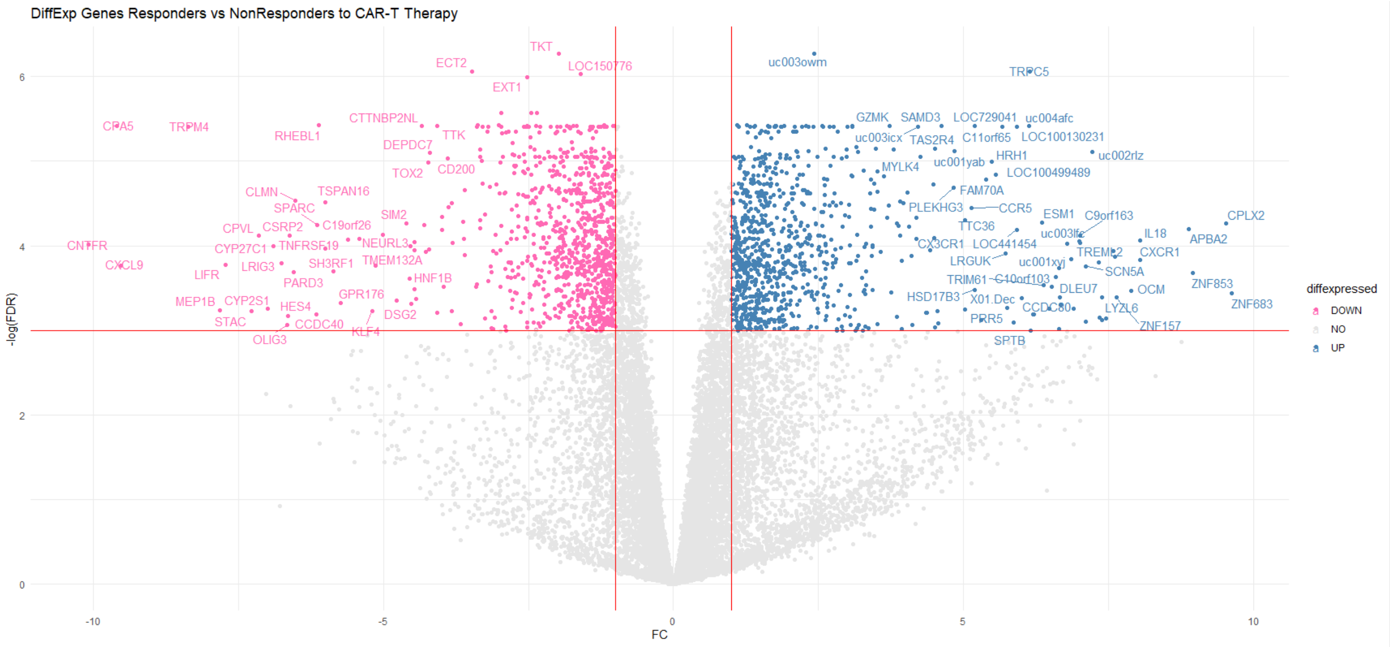

Figure S9. Volcano plot of differentially expressed genes between CR vs. NR groups. False-discovery Rate (FDR) based on moderated t-test in Limma.

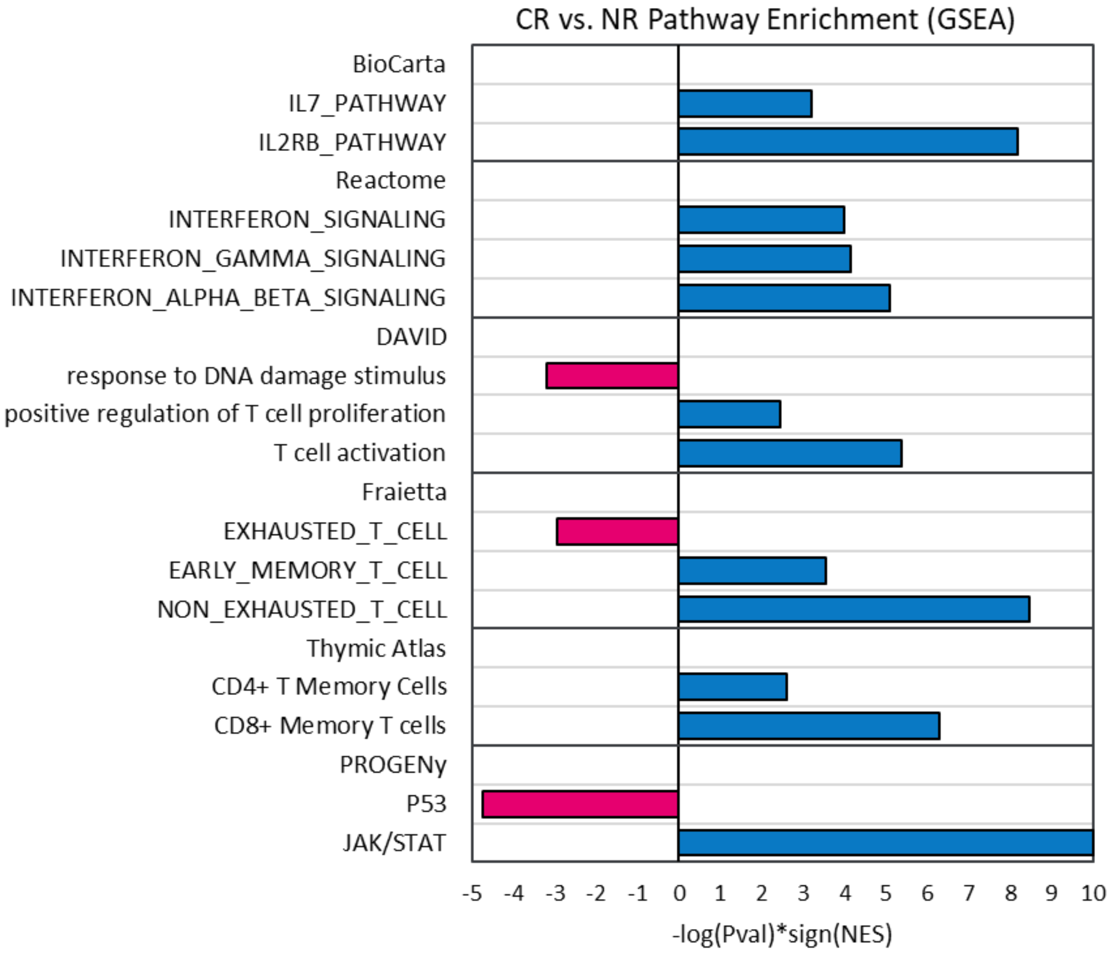

Figure S10. Select gene sets differentially enriched between CR vs. NR groups. Gene sets were derived from BioCarta, Reactome, DAVID, Fraietta et al., Thymic Cell Atlas and PROGENy, and represented as signed $\log_{10}$(P-val) P-values calculated using Kolmogorov-Smirnov test in GSEA.

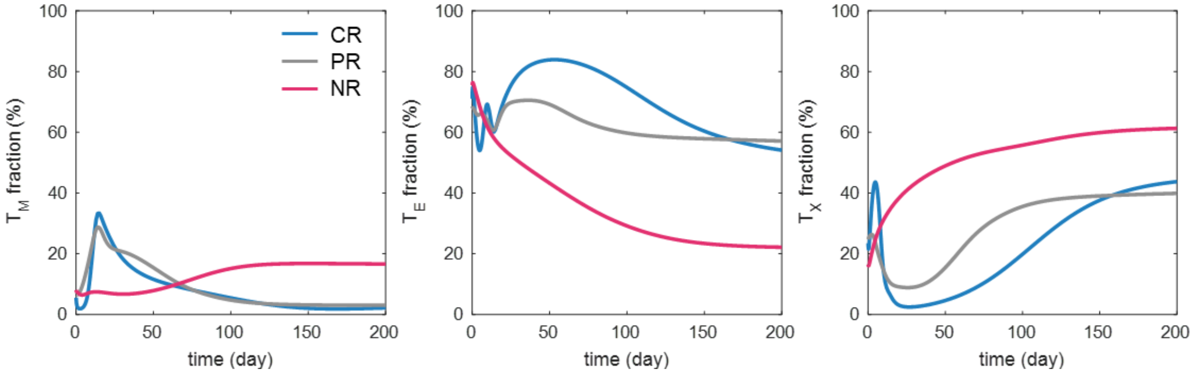

Figure S11. Simulated frequencies of memory (T_M), effector (T_E), and exhausted (T_X) CAR-T cells for CR, PR, and NR patient groups shown in Figure 1.

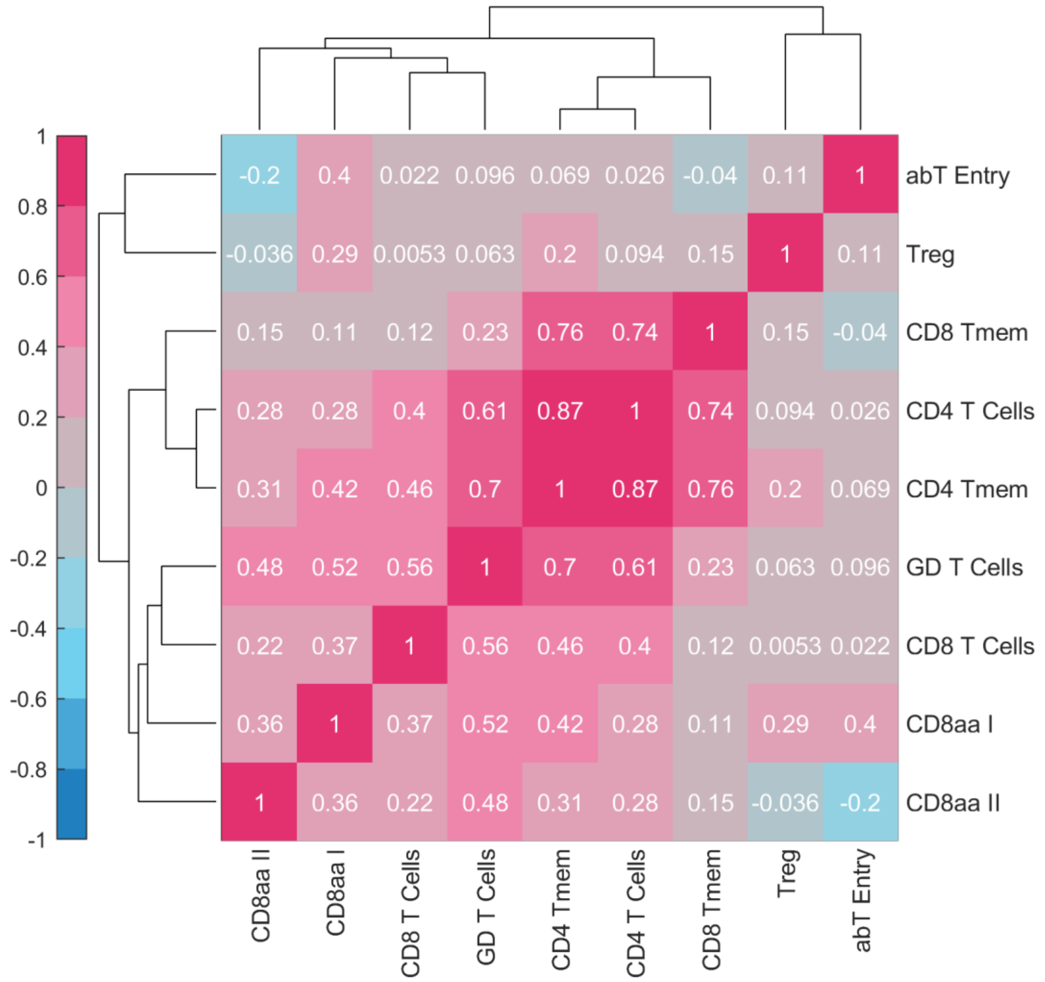

Figure S12. Pairwise Pearson correlation coefficients between Thymic Atlas cell population gene signatures computed using ssGSEA scores from Fraietta et al. RNAseq data. Note signatures for CD8 Tmem, CD4 Tmem, and CD4 T cells are tightly correlated.

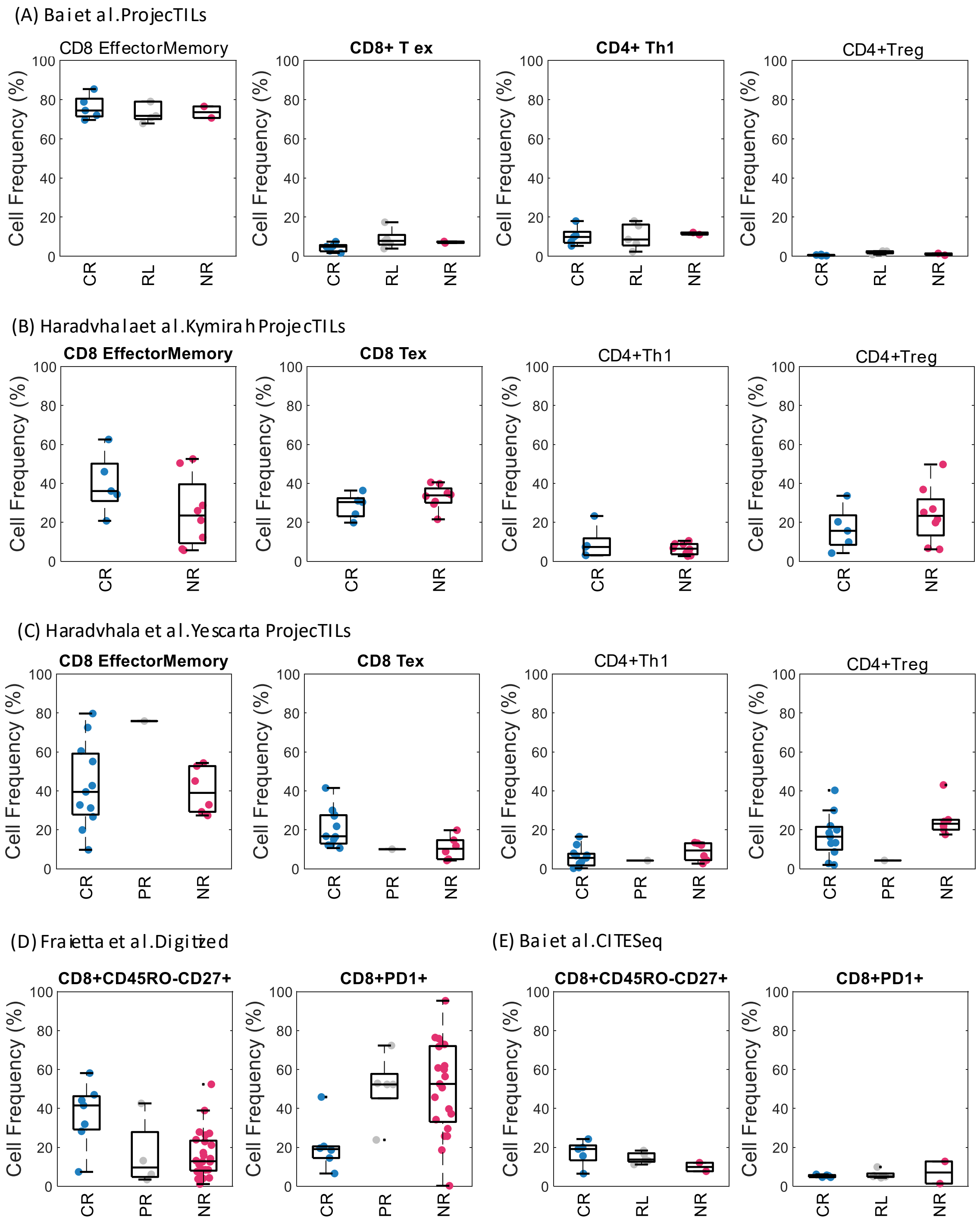

Figure S13: T cell phenotyping of CAR-T infusion products. ProjecTILs-annotated cell frequencies by response category for (A) Kymriah in ALL [22], n=12; (B) Kyrmiach in LBCL [23], n=11; (C) Yescarta in LBCL [23], n=19. Cell types annotated at frequencies of less than 5% are excluded; CD4+ Naïve, CD8+ Naïve, CD8+ Tprecursor-exhausted (Tpex) and CD4+ follicular-helper (Tfh). (D) Immunophenotype-defined T early memory (CD8+CD45RO-CD27+) and exhausted (CD8+PD1+) cell frequencies by response category for Kymriah in CLL, digitized from Fraietta et al.[18], n=38. (B) Immunophenotype-defined Early memory and exhausted cell frequencies by response category in for Kymriah in ALL, calculated from Bai et al.[22] CITEseq antibody tags, n=12. Boxplots represent median ±25 percentiles, and whiskers the min/max value or an additional 1.5-fold quartile distance.

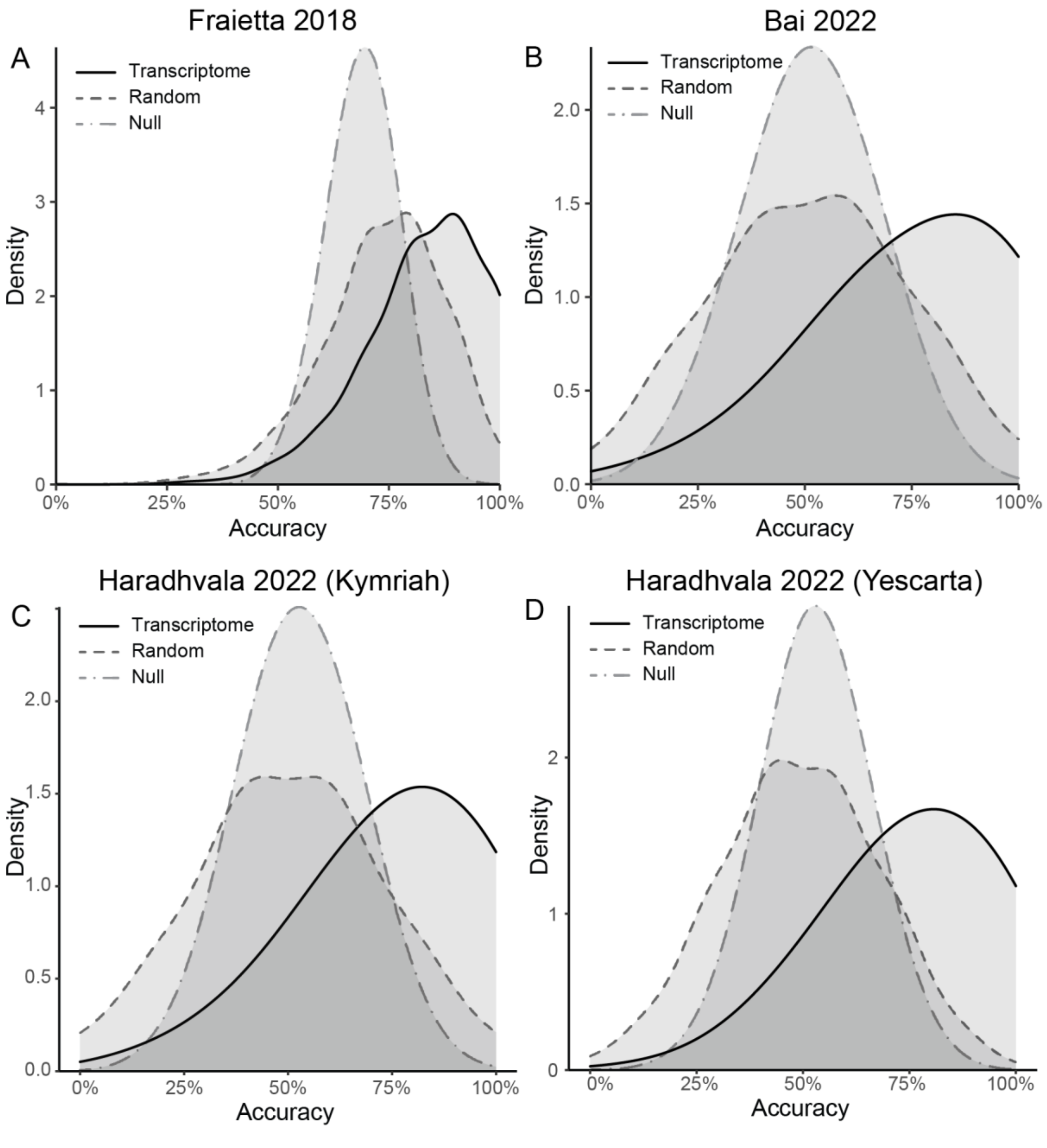

Figure S14: Transcirptome classifier performance as compared to null and random pathway models. Distribution of predictive accuracies are shown for 2500 iterations using 60:40 train:test split cross validation. Results from the 28-signature transcriptome-based ssGSEA classifier ("Transcriptome") are compared null models (random classification; "null") and an ssGSEA classifier trained on a randomized selection of pathways from the compendium ("Random").

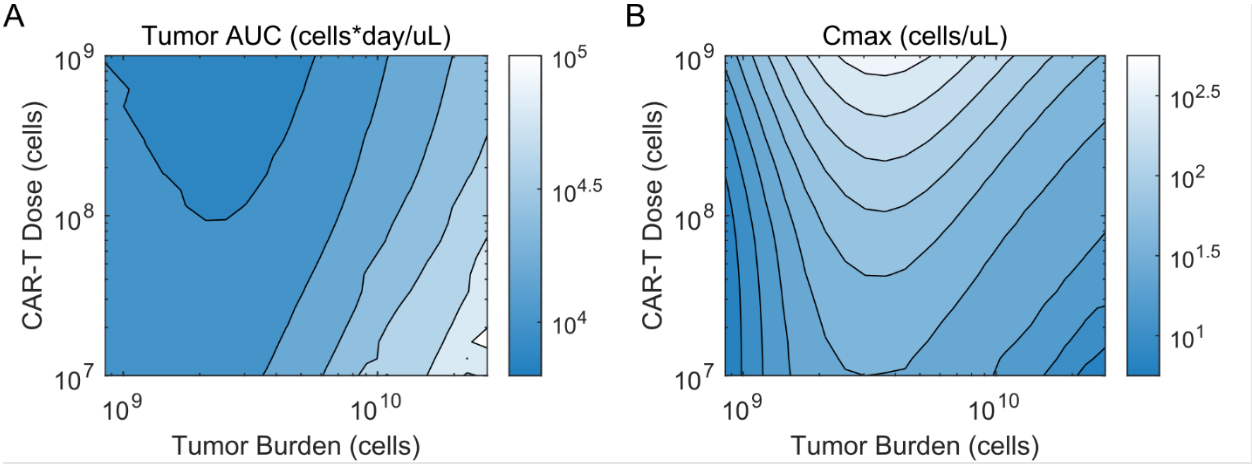

Figure S15: PKPD response depends on initial tumor burden and CAR-T dose. Model simulations were performed across a grid of CAR-T dose, initial tumor burden, and parameter set in the CR population to determine the (A) average tumor AUC and (B) average CAR-T Cmax. Tumor AUC increases with initial tumor burden and decreases with initial CAR-T dose for CR parameters. Cmax exhibits a more complex relationship, peaking for intermediate tumor burdens and generally increasing with initial CAR-T dose. This non-linear interaction between tumor burden and CAR-T dose likely contributes to the clinically observed variability.

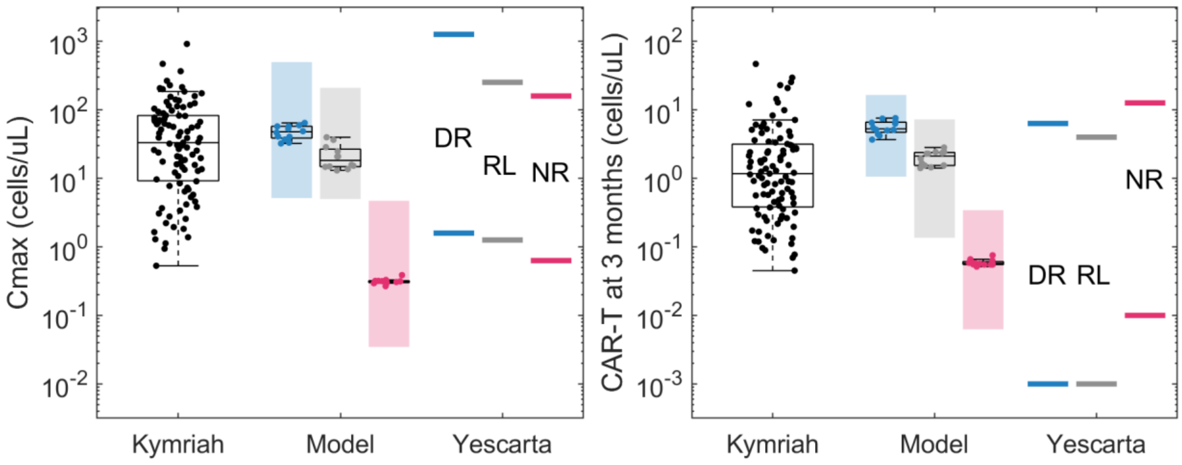

Figure S16: Comparative pharmacokinetics of Kymriah in B-ALL, our model of Kymriah in CLL, and Yescarta in LCBCL. Cmax and C(t =3 month) distributions for Kymriah in B-ALL and CLL were obtained via simulations of the Stein et al. model [2] (n=1000 simulations, represented as percentiles) and our model (n=12 simulations per group), respectively. Data for Yescarta was digitized from Locke et al. [24]. The distributions shown for Kymriah and Model are exactly as in Figure 5C for the left panel (Cmax). The group of boxplots labelled Model show the Cmax or C(t=3 month) for each of the three populations (CR, blue; PR, grey; and NR, pink) with the colored background of the range of Cmax or C(t=3 month) obtained from the clinical PK data. Color bars for Yescarta show digitized min/max ranges of Cmax and C(t=3 month) while the labels (DR, durable response; RL: relapse; and NR: no response) are plotted at the approximate medians. Boxplots represent median ±25 percentiles, and whiskers the min/max value or an additional 1.5-fold quartile distance.

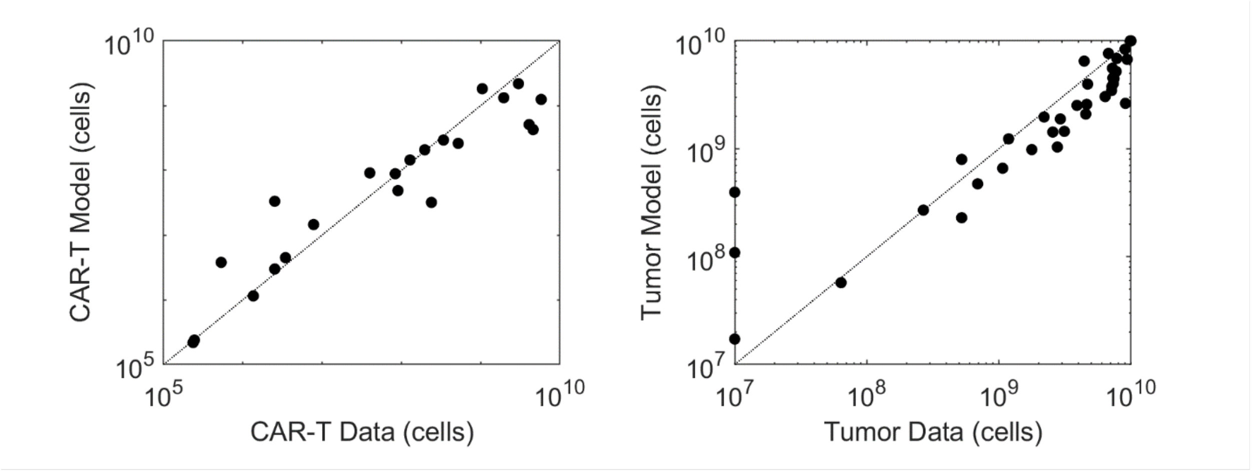

Figure S17: Goodness-of-fit plots to Abecma PKPD data. All data was fit simultaneously, with Pearson's linear correlation coefficient of 0.59 for CAR-T and 0.75 for tumors.

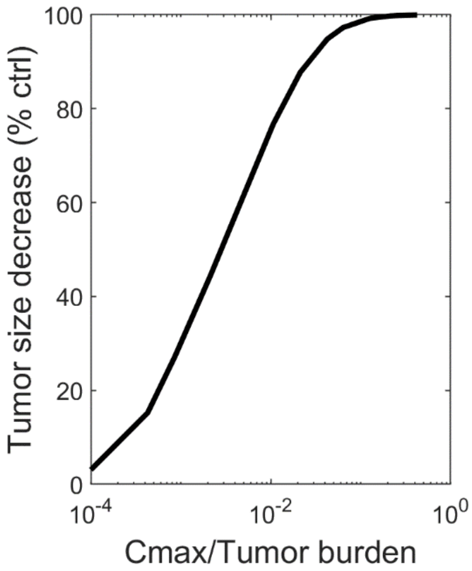

Figure S18. Cmax/Tumor burden vs. tumor response simulations for Abecma. The model fit to Abecma phase1 data was simulated at CAR-T doses ranging from 0.1 to 1000 million cells. Tumor shrinkage, compared to untreated control, was calculated at day 60. The response covariate follows the same trend as that observed for Yescarta in DLBCL and predicted for Kymriah.

REFERENCES

1. Chaudhury, A. *et al.* Chimeric Antigen Receptor T Cell Therapies: A Review of Cellular Kinetic-Pharmacodynamic Modeling Approaches. *J Clin Pharmacol* 60, S147–S159 (2020).

2. Stein, A. M. *et al.* Tisagenlecleucel Model-Based Cellular Kinetic Analysis of Chimeric Antigen Receptor–T Cells. *Cpt Pharmacometrics Syst Pharmacol* 8, 285–295 (2019).

3. Mahlbacher, G. E., Reihmer, K. & Frieboes, H. B. Mathematical Modeling of Tumor-Immune Cell Interactions. *J Theor Biol* 469, 47–60 (2019).

4. Martínez-Rubio, Á. *et al.* A Mathematical Description of the Bone Marrow Dynamics during CAR T-Cell Therapy in B-Cell Childhood Acute Lymphoblastic Leukemia. *Int J Mol Sci* 22, 6371 (2021).

5. Mueller-Schoell, A. *et al.* Early Survival Prediction Framework in CD19-Specific CAR-T Cell Immunotherapy Using a Quantitative Systems Pharmacology Model. *Cancers* 13, 2782 (2021).

6. Hardiansyah, D. & Ng, C. M. Quantitative Systems Pharmacology Model of Chimeric Antigen Receptor T-Cell Therapy. *Clin Transl Sci* 12, 343–349 (2019).

7. Mackey, M. C. Periodic hematological disorders: Quintessential examples of dynamical diseases. *Chaos Interdiscip J Nonlinear Sci* 30, 063123 (2020).

8. Singh, A. P. *et al.* Bench-to-bedside translation of chimeric antigen receptor (CAR) T cells using a multiscale systems pharmacokinetic-pharmacodynamic model: A case study with anti-BCMA CAR-T. *Cpt Pharmacometrics Syst Pharmacol* 10, 362–376 (2021).

9. Singh, A. P. *et al.* Development of a quantitative relationship between CAR-affinity, antigen abundance, tumor cell depletion and CAR-T cell expansion using a multiscale systems PK-PD model. *Mabs* 12, 1688616 (2019).

10. Kimmel, G. J., Locke, F. L. & Altrock, P. M. The roles of T cell competition and stochastic extinction events in chimeric antigen receptor T cell therapy. *Proc Royal Soc B* 288, 20210229 (2021).

11. Hirayama, A. V. *et al.* The response to lymphodepletion impacts PFS in patients with aggressive non-Hodgkin lymphoma treated with CD19 CAR T cells. *Blood* 133, 1876–1887 (2019).

12. Restifo, N. P. & Gattinoni, L. Lineage relationship of effector and memory T cells. *Curr Opin Immunol* 25, 556–563 (2013).

13. Ferreira, D. P. *et al.* Central memory CD8+ T cells derive from stem-like Tcf7 hi effector cells in the absence of cytotoxic differentiation. *Immunity* 53, 985-1000.e11 (2020).

14. Youngblood, B. *et al.* Effector CD8 T cells dedifferentiate into long-lived memory cells. *Nature* 552, 404–409 (2017).

15. Todorov, H. *et al.* CD8 memory precursor cell generation is a continuous process. *Iscience* 25, 104927 (2022).

16. Bresser, K. *et al.* Replicative history marks transcriptional and functional disparity in the CD8+ T cell memory pool. *Nat Immunol* 23, 791–801 (2022).

17. Johnnidis, J. B. *et al.* Inhibitory signaling sustains a distinct early memory CD8+ T cell precursor that is resistant to DNA damage. *Sci Immunol* 6, (2021).

18. Fraietta, J. A. *et al.* Determinants of response and resistance to CD19 chimeric antigen receptor (CAR) T cell therapy of chronic lymphocytic leukemia. *Nat Med* 24, 563–571 (2018).

19. Portet, S. A primer on model selection using the Akaike Information Criterion. *Infect Dis Model* 5, 111–128 (2020).

20. Stein, A. The cellular kinetics and anti-tumor dynamics of Kymriah. in *AcoP10* (2019).

21. Sommer, C. *et al.* Preclinical Evaluation of Allogeneic CAR T Cells Targeting BCMA for the Treatment of Multiple Myeloma. *Mol Ther* 27, 1126–1138 (2019).

22. Bai, Z. *et al.* Single-cell antigen-specific landscape of CAR T infusion product identifies determinants of CD19-positive relapse in patients with ALL. *Sci Adv* 8, (2022).

23. Haradhvala, N. J. *et al.* Distinct cellular dynamics associated with response to CAR-T therapy for refractory B cell lymphoma. *Nat Med* 1–12 (2022) doi:10.1038/s41591-022-01959-0.

24. Locke, F. L. *et al.* Tumor burden, inflammation, and product attributes determine outcomes of axicabtagene ciloleucel in large B-cell lymphoma. *Blood Adv* 4, 4898–4911 (2020).
